# The Use of Dijkstra’s Algorithm in Assessing the Correctness of Imaging Brittle Damage in Concrete Beams by Means of Ultrasonic Transmission Tomography

**DOI:** 10.3390/ma13030551

**Published:** 2020-01-23

**Authors:** Zbigniew Perkowski, Karolina Tatara

**Affiliations:** Faculty of Civil Engineering and Architecture, Department of Physics of Materials, Opole University of Technology, Katowicka 48, 45-061 Opole, Poland; k.tatara@po.edu.pl

**Keywords:** non-destructive testing, ultrasonic tomography, graph theory, concrete, damage mechanics, elastic degradation, damage parameter, internal length, experimental research

## Abstract

The accuracy of transmission ultrasonic tomography for the detection of brittle damage in concrete beams can be effectively supported by the graph theory and, in particular, by Dijkstra’s algorithm. It allows determining real paths of the fastest ultrasonic wave propagation in concrete containing localized elastically degraded zones at any stage of their evolution. This work confronts this type of approach with results that can be obtained from non-local isotropic damage mechanics. On this basis, the authors developed a method of reducing errors in tomographic reconstruction of longitudinal wave velocity maps which are caused by using the simplifying assumptions of straightness of the fastest wave propagation paths. The method is based on the appropriate elongation of measured propagation times of the wave transmitted between opposite sending-receiving transducers if the actual propagation paths deviate from straight lines. Thanks to this, the mathematical apparatus used typically in the tomography, in which the straightness of the fastest paths is assumed, can be still used. The work considers also the aspect of using fictitious wave sending-receiving points in ultrasonic tomography for which wave propagation times are calculated by interpolation of measured ones. The considerations are supported by experimental research conducted on laboratory reinforced concrete (RC) beams in the test of three-point bending and a prefabricated damaged RC beam.

## 1. Introduction

Concrete is one of the most commonly used materials in civil structures. From a scientific and technical point of view, it is a subject of interest both at the stage of designing a recipe, manufacturing various types of elements and during its operation. Ensuring safe and long-term use of concrete structures and elements requires, among other things, appropriate diagnostics. It can use destructive testing (e.g., by testing the strength of drilled cores [1,2]), semi-destructive testing (e.g., pull-out [1,3], pull-off [4,5] methods) and non-destructive testing (NDT, e.g., using sclerometer tests [1,3,6], thermal imaging techniques [7,8], analysis of natural frequencies [9,10], stereological investigations [11], acoustic emission [12,13], X-ray tomography [12], ground-penetrating radars [7,14,15], ultrasound [1,3,7,15,16,17,18,19,20,21,22,23,24,25] including ultrasound tomography [15,19,20,21,22,23,24,25]). The choice of method depends on the material characteristics that we want or are able to measure. All three types of tests are widely known, but especially NDT, thanks to the introduction of a number of modern measurement techniques in the building industry and intensive research, is becoming more and more popular and reliable. Particularly interesting in this area are ultrasound techniques which use at their basis typical phenomena associated with wave motion physics—e.g., reflection, diffraction, attenuation, change of propagation velocity depending on changes in stiffness and density of the medium. The simplest method in the case of concrete structures is an assessment of the velocity of longitudinal waves between selected points of the tested element—the lower the velocity, the lower the stiffness of concrete and its quality [16]. However, it concerns the average speed measurement on the section between the ultrasonic transducers. An interesting, practical case of this type of analysis is article [18] where ultrasonic measurements carried out on a river dam were verified by means of visual inspection of cores taken from it. In the literature, there are also analyses concerning the change of time and intensity of ultrasound wave passing through the area of concrete where a single crack builds up [17]. The use of such research, however, requires in advance knowledge of where such a defect may develop in an element. On the other hand, tomographic methods are deprived of this type of inconvenience, where only a set of transceiver converters suitable for concrete is required and external access to the tested element on one side in a reflective mode (e.g., Reference [21]) or with access on two or more sides in a transmission mode (e.g., Reference [15,20,22,23,24,25]). In the latter view, the state of the material is most often shown indirectly by means of reconstructed maps of the propagation velocity of a selected type of ultrasound wave. For the sake of convenience, longitudinal waves are usually selected for this purpose in the unambiguous interpretation of measurements, as they move the fastest and are not dispersed. It should be emphasized that very accurate maps of cracks in the concrete structure can be made, on the other hand, using X-ray tomography [12]; however, currently, due to the cost of the equipment and the possibility of its use, it is practically impossible to use it directly on real building structures in field research. It is also worth mentioning at this point that, from the point of view of developing mathematical foundations for tomography, its beginning dates back to 1917, when Johann Radon proposed a solution to the problem of reconstruction of the shape of an object on the basis of its projections [26].

The tomographic imaging concrete elements available in the literature focus mainly on the identification of defects with much lower acoustic resistance than the surrounding concrete: e.g., artificially introduced defects, for research purposes, in the form of inclusions from foamed polystyrene [21,22,23], expanded polypropylene [25], prisms from cracked concrete [23] or air-filled pipes [21], cavities in defectively injected pipes for placing prestressing cables [15], areas strongly cracked as a result of excessive loads [20,22,24] or freeze-thaw cycles [19]. Therefore, the first goal that the authors set for themselves in this paper was to carry out an analysis of the extent to which it is possible to detect brittle defects in concrete beams starting from the early stage of their development, when microcracks do not yet form defects capable of effective reflection of waves or their significant slowing down. For this purpose, the methodology of damage mechanics was applied in terms of one of the most recognized concrete models in this field, formulated by Chaboche [27] and Mazars [28], and, in the non-local terms, developed by Pijaudier-Cabot [29,30,31]. Then, depending on the degree of brittle damage described in a “fuzzy” way by the damage parameter, it is possible to model the development of a localized decrease in material stiffness and the associated reduction in the speed of sound waves in concrete. This fact can also be used in the tomographic assessment of concrete [32,33]. For this reason, in order to reliably calculate the distributions of drop in stiffness and changes in the velocity of the longitudinal wave around the forming crack, the authors of the paper proposed an effective way of identifying the parameters of a non-local model of brittle damage evolution using experimental data from [34]. These data were then used in computer simulations of tomographic identification of this type of defect in various phases of its formation and were confronted with the results of our own experimental research.

Another important aspect of ultrasound tomography measurements is its accuracy which may be affected by the diffraction of waves when passing through and around areas of different acoustic resistance than the rest of the medium. It is commonly assumed in order to significantly simplify calculations that paths of the fastest wave propagation are rectilinear (e.g., Reference [15,19,20,21,22,23,24,35]) which is then called rays. This introduces disturbances in tomographic reconstructions when the actual paths differ strongly from the geometry assumed so far. The studies available in the literature show that this assumption does not cause any disturbances in the location of the damaged areas [21,22,23,24]. However, the obtained values of wave propagation velocities on the reconstructed maps differ significantly from the actual values at high levels of concrete degradation [32,33] or inclusions with significantly different acoustic resistance from the matrix [25]. In this case, it should be taken into account that according to the Fermat principle, wave disturbance travels from one point to another such a path that needs minimum or maximum time, or the same in comparison to other, adjacent paths [36], which determines the course of its fastest propagation. As stated in Reference [25], the first attempt to consider the Fermat principle in ultrasound imaging was made by Johnson et al. [37]. They proposed the use of the ray-tracing technique which in the case of concrete structures was first adopted in works [38,39]. An alternative to this type of approach is the use of graph theory, in particular Dijkstra’s algorithm [40]. If we apply it in the analyzed issue to a full graph the nodes of which will cover the studied area, and the weights of the edges will be equal to the time of the wave passing through them, then in this way we can approximately determine the shape of paths of the fastest sound propagation and the time needed for the wave to travel along them. The results will be the more accurate the denser the network of nodes. This very interesting concept, inspired by the works of various authors and applied to seismic waves, was developed in Moser’s work [41] in 1991 (as in Reference [25]). Extensive studies in this field with examples of calculations using experimental data from concrete cubes with inclusions from expanded polypropylene have been presented in Reference [25]. A proposal to use this methodology in the ultrasonic testing of structural elements was also presented by the authors in Reference [33,42]. In the light of the quoted information, it can be noted that so far there is no analysis in the literature concerning this type of problem in the case of tomographic assessment of the damage evolution described according to the concept of damage mechanics. Therefore, the second aim of this article was to present in this area appropriate numerical analyses with the use of Dijkstra’s algorithm in determining shape of the paths of the fastest ultrasound wave propagation. On this basis, the author’s own method of improving the accuracy of tomographic calculations was formulated because of the inconsistency of the assumptions with reality in the case of using straight-line rays to approximate the geometry of the fastest propagation paths. For this purpose, it is proposed to not interfere with the assumption of straightness of ultrasound pulse pathways but, on the other hand, to properly scale the measured times of their propagation between the assumed transceiver points elongating them in the case of rays that pass through elastically degraded areas. The advantage of such an approach is that it does not complicate well established mathematical methods (e.g., Reference [35,43,44,45,46]) which have been implemented in tomographic imaging techniques.

The paper also raises the practical aspect of conducting such measurements by analyzing in the light of the presented arguments how the accuracy of results may be affected by the introduction of the so-called fictitious transceiver points for which the times of propagation of ultrasound waves are interpolated on the basis of measurements from the real points. In this respect, the authors were inspired by work [47] where this approach was presented in the study of moisture distribution in walls. It may significantly reduce the number of points used and the labor intensity in real measurement situations, which is particularly important in the case where fewer ultrasonic transducers are available.

Due to the scope of studies within the framework of the presented calculation examples and experiments, the authors limited themselves to the case of concrete beams with elastically degraded zones perpendicular to the beam axis. All the calculations made in the article were done with the use of the authors’ computer programs written in the MATLAB software environment.

## 2. Ultrasonic Transmission Tomography

**Figure 1:**
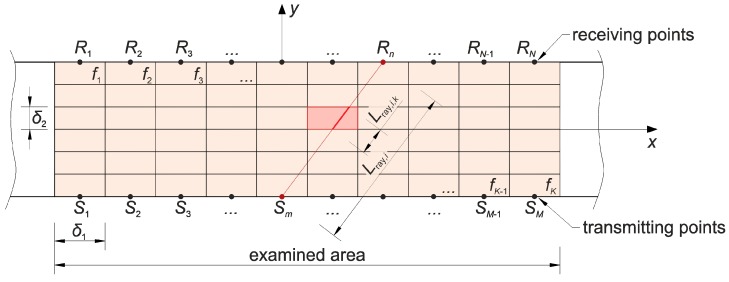
Scheme of a cell system, transmitting/receiving points, and rays in a plane area examined tomographically.

The considerations presented in the paper concern the case of concrete beams that contain elastically degraded zones (in the form of grouped micro-cracks or cracks) running across the entire cross-section—e.g., as a result of simple bending or tension. Therefore, tomographic analyses were narrowed down to the identification of brittle damage in a flat longitudinal section of beam which will also be the plane of symmetry of this element, focusing on the assessment of changes in the speed of ultrasonic waves due to a local change in the stiffness of the cracked material. For this reason, in all computational examples and experimental studies presented in the paper, a system of opposite transmitting/receiving points of ultrasound waves in the transmission mode was used (Figure 1). Longitudinal waves have been selected as non-dispersive and the fastest of all ultrasonic wave types for the study. This requires, however, that their length should be small enough in relation to the dimensions of the cross-section of the element to be effectively generated (practical recommendations in this respect can be found in, e.g., Reference [48]). On the other hand, it limits the distance between the intended transmitting/receiving points because of the attenuation of the longitudinal waves and the angle at which they may propagate from the transmitting point in an effective manner in reception because of the amplitude distribution. In the latter case, on the basis of individual experiences of the authors, the angle of inclination of diagonal rays to a bar axis was limited to range from 45° to 135°; in Reference [33], a numerical simulation of the process of propagation of ultrasounds during its excitation by the transducer was performed where it was shown that the amplitudes of longitudinal waves are negligible in contrast to transverse waves outside this angular range, which may result in incorrect and increased propagation time reading.

From the mathematical point of view, by indirect visualization of the material structure by means of maps of distributions of quantities characterizing the propagation of ultrasound waves, it is necessary to build an appropriate system of equations (e.g., Reference [35]). The plane problem assumes that the reconstructed image consists of a finite number of plane cells which, in the examined area, is separated by an orthogonal grid of a step of $\delta_{1}\times \delta_{2}$ (Figure 1). The function is searched for in an approximate way:

(1) $$f=c_{L}^{-1}\left(x,y\right),$$

where: $c_{L}$—longitudinal wave velocity (m/s); x, y—spatial variables (m). For this purpose, it is assumed that f in each cell takes a constant value of $f_{k}$ where k=1,2,…,K.

In the problem, the longitudinal wave propagation times between the selected sending ($S_{m}$) and receiving ($R_{n}$) points must be given where m=1,2,…,M; n=1,2,…,N. It is assumed to simplify considerations that the fastest propagation path between these points can be modeled as a straight line, which in tomography is referred to as a ray. The propagation time of $t_{\mathrm{ray},i}$ (s) over the i-th ray, connecting points $S_{m}$ and $R_{n}$, can be calculated from the integral:

(2) $$t_{\mathrm{ray},i}=\int_{S_{m}}^{R_{n}}f\left(l_{i}\right)dl_{i},$$

where: $l_{i}$—variable describing the position on the i-th ray (m); i=1,2,…,I. Considering that averaged values f are being sought in the cellular areas, we can write that:

(3) $$t_{\mathrm{ray},i}=\sum_{k=1}^{K}L_{\mathrm{ray},i,k}f_{k}\to Ax=b,$$

where: $L_{\mathrm{ray},i,k}$—parts of the length of the i-th ray falling on the k-th cell (m) (if the ray does not pass through cell k, then $L_{\mathrm{ray},i,k}=0$), $A={\left[L_{\mathrm{ray},i,k}\right]}_{I\times K}$ (m), $x={\left[f_{1},f_{2},\ldots ,f_{K}\right]}^{T}$ (s/m), $b={\left[t_{\mathrm{ray},1},t_{\mathrm{ray},2},\ldots ,t_{\mathrm{ray},I}\right]}^{T}$ (s). The above is the definition of a system of equations in relation to the value of $f_{k}$, which is used to determine the velocity distribution of ultrasound wave propagation in tomography. After its solution, we get that:

(4) $$c_{L\;\mathrm{tom},k}=\frac{1}{f_{k}},$$

where $c_{L\;\mathrm{tom},k}$—mean velocity of the longitudinal wave in the k-th cell (m/s) (in the sense of the presented method). Please note that I (the number of rays used) must not be less than K (the number of the assumed cells) and the rays must evenly cover the test area. The system of equations formulated in such a way is an ill-conditioned one, which forces the use of iterative techniques of its solving. The basic method in this respect is an algorithm developed by the Polish mathematician Stefan Kaczmarz (1937). In 1970, Gordon and his collaborators, working on the application of this technique in medicine, rediscovered this method and named it Algebraic Reconstruction Technique (ART) [43]. It was this one that was used in the first in the world computed tomography scanner constructed by Hounsfield in 1972 [44]. On the basis of the Kaczmarz algorithm, many other methods were developed. Currently, the literature distinguishes three basic ways of imaging: the aforementioned ART, Simultaneous Iterative Reconstruction Technique (SIRT), and Simultaneous Algebraic Reconstruction Technique (SART) which is a combination linking the advantages of the ART and SIRT methods [35,46]. For this purpose, the Tikhonov regularization method can also be used in the method of least squares (e.g., Reference [24]).

In this article, all tomographic images presented below were solved with the use of a randomized Kaczmarz algorithm. The final result was taken as $x_{\mathrm{mean},q}$, i.e., the mean of q independently obtained solutions of equation system (3). Hence:

(5) $$x_{\mathrm{mean},q}=\frac{1}{q}\sum_{r=1}^{q}x_{r}.$$

In a given solution $x_{r}$, its subsequent approximations were made by projecting the previous approximation in a direction perpendicular to the randomly selected straight lines defined by the equations of system (3), but so that each of these lines would be used only once. The starting point for the iteration of each $x_{r}$ was the vector:

(6) $$x_{0}={\left[c_{L\;\mathrm{ref}}^{-1},c_{L\;\mathrm{ref}}^{-1},\ldots ,c_{L\;\mathrm{ref}}^{-1}\right]}_{1\times K}^{T},$$

where $c_{L\;\mathrm{ref}}$—reference value of the longitudinal wave velocity (m/s). The number q of the averaged solutions of equation system (3) were selected so that the condition was met:

(7) $$\left|\frac{e_{g,q}-e_{g,q-1}}{e_{g,q}}\right|<10^{-5}\;\mathrm{and}\;e_{g,q}=\frac{\left||Ax_{\mathrm{mean},q}-b|\right|}{\left||b|\right|}.$$

As mentioned in the introduction, another inconvenience of the presented method of tomographic imaging is the fact that ultrasonic waves are diffracted when avoiding areas with different acoustic resistance, so that the real paths of the fastest propagation are curvilinear. This is one of the basic sources of inaccuracy of the presented approach if there are sub-areas with significantly different acoustic resistances in the tested concrete element in relation to the rest of the element [25,32,33]. These issues will be discussed in the context of the following calculation examples and experimental studies concerning the detection of elastically degraded concrete zones.

## 3. Localized Elastic Degradation in Concrete—Crack Model in Concrete According to the Damage Mechanics

**Figure 2:**
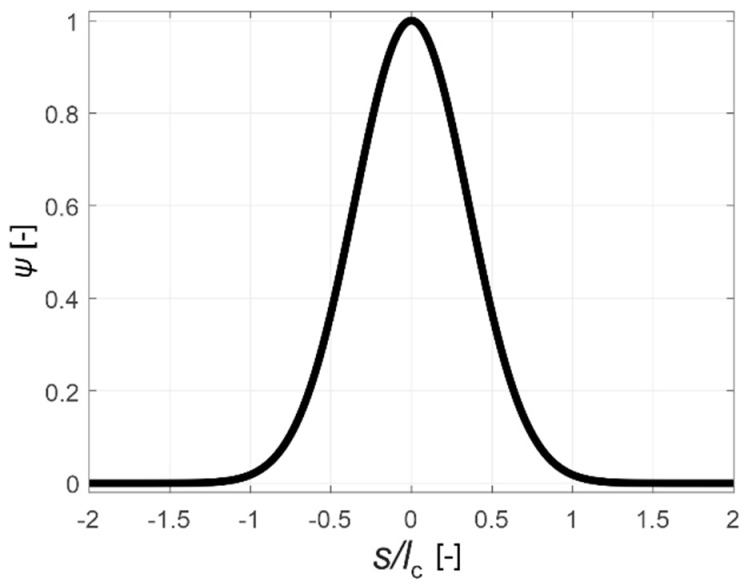
Distribution of the weight function ψ for x=0.

In concrete, due to its brittleness, the evolution of damage occurs in particular at the action of tensile stresses [27,28,29,30,31]. The process begins with the formation of microcracks which, with further increase in stress, grow into localized damage zones and cracks visible to the naked eye. For this reason, the presence of such zones at the final stage can be easily detected visually, as they contain cracks of the order of tenths of a millimeter in width. On the other hand, they are not visible at an early stage of development, and what is important, the initial microcracks usually do not occur in random places of concrete structure. When the cementitious material structure develops, the high and low internal cohesion zones can be distinguished. Especially, the places with low cohesion are the ones where cracks begin to grow because, in such places, even a small amount of released energy causes their propagation. They form mainly around the micro-pores or near the phase separation surfaces [11]. From the analyses presented in this respect within the damage mechanics, it is known that this process obviously leads to elastic degradation of concrete, which means a local decrease in material stiffness [27,28,29,30,31,49]. This phenomenon gives grounds for detection and control of this type of damage by ultrasound tomography if the spatial distribution of propagation velocity of a selected type of mechanical waves (e.g., Reference [24,33]) is assessed within such a framework. That is why, in the article, the preliminary calculations were oriented on determining the spatial distribution of concrete stiffness change in the case of localized damage under tension. The necessary information was obtained in this way in order to analyze the propagation of ultrasound waves in a localized elastically degraded concrete zone which evolution leads finally to forming macro-cracks. The calculations were performed using the assumptions of one of the most popular models in this respect, introduced by Chaboche [27] and Mazars [28], which takes into account the weakening of the material due to the isotropic damage accumulation and which was developed by Pijaudier-Cabot [29,30,31] in non-local terms. In a uniaxial tensile state, the model assumes the following relationship between stress and deformation (using the principle of strain equivalence):

(8) $$\sigma =\left(1-D\right)E_{0}\varepsilon ,$$

where: σ—tensile normal stress (Pa); ε—normal strain [-] $E_{0}$—Young’s modulus in undamaged material (Pa); D—damage parameter [-]. Due to thermodynamic limitations of the process, the following must be satisfied:

(9) $$\mathrm{if}\;f_{D}=\bar{\varepsilon }-\kappa =0\;\mathrm{and}\;\dot{\bar{\varepsilon }}>0,\;\mathrm{then}\;D=1-\left(1-A_{t}\right)\frac{\kappa_{0}}{\kappa }-A_{t}\;\exp\left(-B_{t}\left(\kappa -\kappa_{0}\right)\right)\;\mathrm{and}\;\dot{\kappa }=\dot{\bar{\varepsilon }},$$

(10) $$\mathrm{if}\;f_{D}=\bar{\varepsilon }-\kappa <0\;\mathrm{or}\;\dot{\bar{\varepsilon }}\leq 0,\;\mathrm{then}\;\dot{D}=0\;\mathrm{and}\;\dot{\kappa }=0,$$

where: $A_{t}$, $B_{t}$—material parameters [-]; $f_{D}$—load function [-]; κ—variable describing the process of material weakening [-]; $\kappa_{0}$—initial value of the variable κ [-]; $\bar{\varepsilon }$—non-local equivalent tensile strain [-]. To simplify the problem, if we assume that pure tension occurs in a concrete element with its axis described by variable x (m), the non-local equivalent strain at point x will be defined as:

(11) $$\bar{\varepsilon }\left(x\right)=\frac{1}{V_{r}}\int_{s_{\left(1\right)}}^{s_{\left(2\right)}}\tilde{\varepsilon }\left(s\right)\;\psi \left(x-s\right)A\left(s\right)\;ds,$$

(12) $$V_{r}\left(x\right)=\int_{s_{\left(1\right)}}^{s_{\left(2\right)}}\;\psi \left(x-s\right)A\left(s\right)\;ds\;,$$

(13) $$\psi \left(x-s\right)=\exp\left(\frac{-4{\left(x-s\right)}^{2}}{l_{c}^{2}}\right),$$

(14) $$\tilde{\varepsilon }=\sqrt{\sum_{i=1}^{3}{\langle \varepsilon_{i}\rangle }^{2}},$$

where: $\tilde{\varepsilon }$—local equivalent tensile strain [-]; $\varepsilon_{i}$—local principal strain [-]; $V_{r}$—representative volume of the material (m^3^), s—variable describing the position along axis x (m); $s_{\left(1\right)}$, $s_{\left(2\right)}$—starting and ending point of the considered element (m); A—cross-sectional area of the element (m^2^); $l_{c}$—the characteristic dimension of the non-locality or the so-called internal length (m); ψ—weight function [-]; 〈…〉—Macauley’s operator. Variability of function ψ is adopted in the model identical to the normal distribution with standard deviation $\frac{l_{c}}{2\sqrt{2}}$. It also means that the range of the non-locality practically ceases to be significant above the distance $l_{c}$ because $\psi \left(x-s=l_{c}\right)=1.83\cdot 10^{-3}$ (Figure 2). In addition, in the case of uniaxial tension, $\tilde{\varepsilon }=\varepsilon$ where ε is the normal strain along the axis of the beam, $\kappa_{0}$ will be equal to this deformation at the moment of initiation of the damage evolution, and $\kappa_{0}$ can reach a maximum value of $f_{t}/E_{0}$ where $f_{t}$ is the tensile strength. In the incremental version needed for the numerical analysis of the problem, the stress and damage evolution Equations (8)–(10) take the following forms:

(15) $$\mathrm{if}\;f_{D}=\bar{\varepsilon }-\kappa =0\;\mathrm{and}\;\Delta \bar{\varepsilon }>0,\;\mathrm{then}\;\{\begin{array}{c} \Delta \sigma =\left(1-D\right)E_{0}\Delta \varepsilon -\Delta DE_{0}\varepsilon \\ \Delta D=\frac{\partial D}{\partial \bar{\varepsilon }}\Delta \bar{\varepsilon }=\frac{\partial D}{\partial \bar{\varepsilon }}\frac{1}{V_{r}}\int_{s_{\left(1\right)}}^{s_{\left(2\right)}}\Delta \tilde{\varepsilon }\left(s\right)\;\psi \left(x-s\right)A\left(s\right)\;ds\\ \Delta \kappa =\Delta \bar{\varepsilon } \end{array},$$

(16) $$\begin{array}{c} \mathrm{if}\;f_{D}=\bar{\varepsilon }-\kappa <0\;\mathrm{or}\;\Delta \bar{\varepsilon }\leq 0,\;\mathrm{then}\\ \;\{\begin{array}{c} \Delta \sigma =\left(1-D\right)E_{0}\Delta \varepsilon \\ \Delta D=0\\ \Delta \kappa =0 \end{array}, \end{array}$$

where: Δ…—the finite increment of a given quantity.

**Figure 3:**
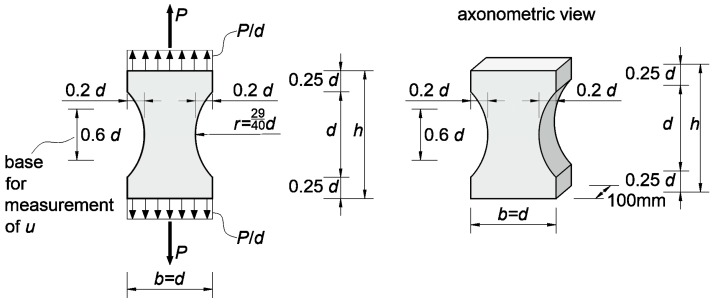
Shape of concrete specimens for the tension tests (based on Reference [34]).

**Figure 4:**
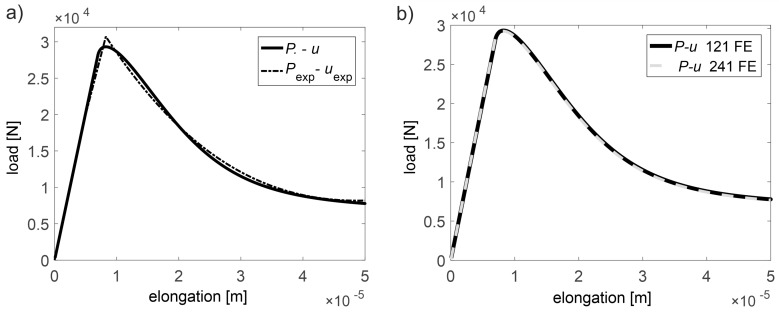
(**a**) Comparison of relations $P_{\exp}$-$u_{\exp}$ and P-u using 121 finite elements. (**b**) Comparison of relations P-u using 121 and 241 finite elements.

A separate issue related to the use of the discussed model is the adoption of appropriate material parameters that will enable the most accurate capture of the real behavior of concrete. The authors in paper [31] give the approximate typical ranges of these parameters in case of concrete of moderate strength, i.e., $E_{0}=30–40$ GPa, $A_{t}=0.7–1.2$, $B_{t}=10^{4}–5\cdot 10^{4}$, $\kappa_{0}=10^{-4}$, and $l_{c}=3–5\;d_{a\;\max}$ where $d_{a\;\max}$ is the maximum size of the aggregate. However, so far, there are no exhaustive items in the literature devoted to research and formulation of automatic optimization calculation techniques allowing for precise estimation of all parameters mentioned above for a given type of concrete due to the length $l_{c}$. It is assessed using mainly the scale effect and the model is calibrated by a manual trail-and-error method (e.g., Reference [31,49,50]). For this purpose, a suitable coefficient inverse problem is formulated in this article which uses illustratively the data from [34]. These tests concern the uniaxial tensile testing of a series of “dog bone” shaped concrete specimens of mean compressive strength $f_{\mathrm{cm},\mathrm{cube}}=50$ MPa and $d_{a\;\max}=8$ mm. The scheme of specimens is shown in Figure 3, and the experimental load-elongation dependencies ($P_{\exp}$-$u_{\exp}$) of a selected series of specimens of an overall length of 30 cm within a range of up to 50 μm are shown in Figure 4a. This relation was obtained on the basis of digitalization of graphs presented in Reference [34], and due to their quality by entering the curve in the middle area formed by the envelope of all several curves measured on the specimens of the length of 30 cm. For calculations, data from this series of samples were selected from all 7.5 cm, 15 cm, 30 cm, 60 cm, 120 cm, and 240 cm length series, which were tested in Reference [34], because in their case the relatively highest number of measurements was obtained with possibly small scatter of measured tensile strength. On the other hand, samples with a length of 7.5 cm were characterized by the largest scatter of measurement results on the basis of which the authors of [34] concluded that the width of their smallest section is smaller than the length $l_{c}$ and it can be larger. For this reason, its value was suggested as $6–7\;d_{a\;\max}$. The elongation measurement base was 0.6 times the notch length in the specimen (Figure 3). It should be noted at this point that a very interesting analysis of the development of elastically degraded zones based on the data from work [34] was also presented in Reference [50] but without formulating a coefficient inverse problem.

The tests included in Reference [34] were also selected for analysis due to the fact that the shape of the specimens enables, in tension, the location of the brittle damage zone in their middle area, leading to the formation of a single macro-crack when the load capacity is exhausted. In the considerations, for the sake of simplification, a bar in tension of variable cross-section was used as a model of specimens, according to their geometrical features. This choice was dictated by the need to carry out the calculation procedures described below in an acceptable time frame due to the computer hardware available (PC with a processor 3.06 GHz and RAM 8 GB—time of solving one task approximately 2 min). The boundary problem analyzed was solved using the finite element method (FEM) in an incremental version using physical Equations (15),(16). Two-node bar finite elements with 2 degrees of freedom and constant and averaged over the length physical and geometric features (with one integration point in the middle of the element) were used. One hundred and twenty-one finite elements were adopted on the axis of the specimen of the length of 30 cm. In addition, the load increments of the specimen were assumed to be continuously elongated during the calculation, which meant switching the force sign at the transition to the weakening phase. The force increment, in turn, was selected in each step so that the increments in elongation of the measuring base and normal strain in each of the elements would not exceed, respectively, the values of 0.15 μm and $1.25\times 10^{-6}$. Taking into account the above mentioned conditions of calculations allowed to obtain a satisfactory convergence of the solution. Further increase of the number of elements and decrease of permissible increments of strain and displacements did not significantly affect the result (Figure 4b).

First, $E_{0}$ was estimated by adjusting the slope of the initial straight-line section to the measuring point $\left[P_{\exp},u_{\exp}\right]$ = [20 kN, 5 μm] in the load-elongation relation (P-u) obtained from the model in the elastic range. The calculus procedure in this case was realized using ordered domain search. Hence, it was determined that $E_{0}=38.29$ GPa. Other parameters, i.e., $A_{t}$, $B_{t}$, $\kappa_{0}$, and $l_{c}$ were estimated by minimizing the objective function:

(17) $$F\left(p\right)=\sum_{i=1}^{n}{\left(P_{i}\left(p\right)-P_{\exp,i}\right)}^{2},$$

where: $P_{\exp,i}$—specimen loads from the experiment (N) determined in the weakening phase (Figure 4a) for elongations at equidistant intervals starting from $u_{\exp}=8.25$ μm (for $P_{\exp}=P_{\max}=30.72$ kN—i.e., maximum load) to $u_{\exp}=50$ μm (for $P_{\exp}=8.18$ kN); $P_{i}$—equivalents $P_{\exp,i}$ determined on the basis of the assumed model (N); $p=\left[p_{1},p_{2},p_{3},p_{4}\right]$—vector of variables corresponding to the parameters searched for, i.e., $A_{t}$, $B_{t}$, $\kappa_{0}$, and $l_{c}$, respectively. On that basis, it was assumed that:

(18) $$\;\arg\;\min\;F\left(p\right)=\left[A_{t},\;B_{t},\kappa_{0},l_{c}\right].$$

**Figure 5:**
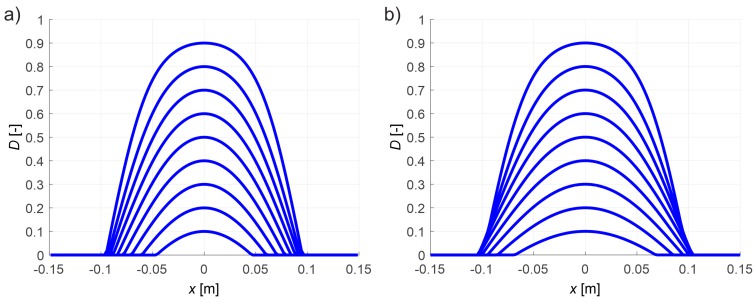
Calculated distributions of D during the development of the damaged zone in the middle of the specimen: (**a**) model of the specimen d×h=20 cm × 30 cm (**b**) specimen model with twice the total width and length increased d×h =40 cm × 60 cm (d, h according to Figure 3).

The calculations in formula (17) assumed n=31. The minimization of the implicit function (17) was carried out in three stages. In the first stage, a genetic algorithm was used on a population of 20 parameter vectors p in 20 selections. The vector components of the first population were drawn in preselected intervals: $p_{1}$ of [0.7, 1.2], $p_{2}$ of [$10^{4},5\times 10^{4}$], $p_{3}$ of $\left[5\times 10^{-4},P_{\max}/(E_{0}A_{\min})\right]$ and $p_{4}$ of $\left[3\;d_{a\;\max},11\;d_{a\;\max}\right]$, where $A_{\min}$ [m^2^] is the minimum cross-sectional area of the specimen at the center of its length; hence, $P_{\max}/A_{\min}=f_{t}$. It should be noted that the upper limit of interval selected for $p_{4}$ is greater than that given in Reference [31,34]. The authors assumed finally the value $11\;d_{a\;\max}$ as the first smallest one for which the genetic algorithm did not estimate placing the minimum point of F right next to the upper limit of $p_{4}$. Subsequent generations of the population were created as follows: p with the lowest value of F passed unconditionally to the next generation, the next 10 new vectors p were obtained by arithmetic crossover of randomly selected p from the group of the first 14 vectors of the old generation after their ordering from the lowest to the highest value of F. The set of the last nine new vectors p were drawn in the same way as in the first generation. This procedure was repeated independently five times. In the second stage, the same procedure as in the first stage was followed, however, changing the draw intervals of vector p components. The boundaries of them were defined from 0.8 to 1.2 of the the values of the p vector components for which the lowest value of F was obtained in stage one. In the third and final stage, an orderly search of the domain of acceptable solutions was performed in the vicinity of the point defined by the vector p components with the lowest F-value found in the second stage. The search interval was selected from 0.99 to 1.01 of the values of the parameters of this vector. If the minimum F in this area was at the boundary of any interval, the procedure was repeated where the point with the current minimum value F determined the midpoint of the intervals in the next step. In this way, the following values of the parameters of the concrete damage evolution equations were obtained: $A_{t}=8.01\times 10^{-1}$, $B_{t}=1.95\times 10^{4}$, $\kappa_{0}=5.64\times 10^{-5}$ and $l_{c}=112$ mm while the objective function F reached the value $6.07\times 10^{6}$ N^2^. This outcome corresponds to the global mean square relative error $\surd (\;\sum_{i=1}^{n}{\left(P_{i}\left(p\right)-P_{\exp,i}\right)}^{2}/\sum_{i=1}^{n}P_{\exp,i}{}^{2})=0.027$ which expresses matching the experimental curve to the theoretical one in the weakening phase. In Figure 4a, the experimental curve and model one for the determined parameters were compared. Figure 4b also shows the course of the model curve after increasing the number of elements to 241 and reducing the permissible increments of measurement base elongation and normal strain to the value of 0.075 μm and $6.25\times 10^{-7}$, respectively, to confirm the correctness of the calculations from the point of view of ensuring the convergence of the solution. In this case, the mean square relative difference between the solutions in the weakening phase with dense and rare discretization amounted to $\surd (\;\sum_{i=1}^{n}{\left(P_{241,i}-P_{121,i}\right)}^{2}/\sum_{i=1}^{n}P_{121,i}{}^{2}\;)=7.6\times 10^{-3}$ where in the subscripts the number of elements used is given. On the other hand, Figure 5a shows the distribution of the damage parameter D at its different maximum values along the axis of the specimen at its middle section during the growth of the localized damage. It is also an equivalent way of modeling the development of macro-crack formation from the point of view of damage mechanics [30,49]. The D-distributions with a proportional 2-fold increase of all dimensions of the specimen model (Figure 5b) with the same values of parameters determining the accuracy of the solution were also calculated in a comparative way. A very similar variability of individual distributions was obtained, while the maximum width of the equivalent macro-crack zone reached a value approximately equal to $2l_{c}$. This confirms the correctness of the presented method of modeling the localized damage evolution in the case of tension in concrete. The presented considerations also justify the adoption of a specific resolution in tomographic imaging by means of wave velocity maps, i.e., dimensions $\delta_{1}$ and $\delta_{2}$, according to Figure 1. In an extreme case, they should not be greater than $2l_{c}$, however, in order to ensure adequate image sharpness and precise identification of the degree of damage, they should be adequately smaller. Taking into account the variability of the distribution D, it is reasonable that $\delta_{1}$ and $\delta_{2}$ were assumed in the interval of approximately $l_{c}/3-l_{c}/5$. The presented result also shows that the knowledge of the value of $l_{c}$ is crucial for the correct assessment of the distribution of damage around the crack in ultrasound tomography, and, on the other hand, it is the tomography that can be used for the direct assessment of this value.

**Figure 6:**
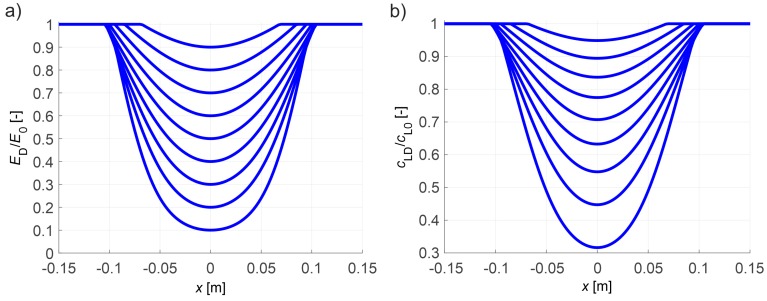
Relative changes of Young’s modulus (**a**) and longitudinal wave velocities (**b**) for distributions of D from Figure 5b.

Given that the growth D according to relation (8) causes the decrease of Young’s modulus of the material, i.e.,:

(19) $$\frac{E_{D}}{E_{0}}=1-D,$$

where: $E_{D}$—tangential Young’s modulus (Pa) of the damaged material during inactive growth of the damage, it is in its micro-cracked zones that the velocity of ultrasound waves decreases. Hence, based on the simplified isotropic damage evolution model for concrete developed in Reference [27,28,29,30,31] for three-dimensional stress state and assuming negligible change in material density during the evolution of brittle damage, the following estimated relationships can be given:

(20) $$\frac{c_{\mathrm{LD}}}{c_{L0}}=\sqrt{\frac{E_{D}}{E_{0}}}\;\mathrm{or}\;\frac{E_{D}}{E_{0}}={\left(\frac{c_{\mathrm{LD}}}{c_{L0}}\right)}^{2},$$

where: $c_{L0}$, $c_{\mathrm{LD}}$—the velocity of the longitudinal wave in the virgin and damaged material, respectively, (m/s). Based on relations (19), (20), variabilities of Young’s modulus and longitudinal wave velocity are shown in Figure 6 which correspond to the damage parameter distributions from Figure 5b. They will be used in the computational examples presented in point 4 that illustrate the problem of tomographic detection of cracks in concrete members at various stages of their evolution.

It needs to be highlighted that the numerical results presented in this point can be directly used only in the case of pure tension in concrete. However, they can be also generalized usefully for the case of bent reinforced concrete (RC) beams when estimating the damage level in concrete of such beams with the following simplifying assumptions: the planar cross-sections of RC beam remain planar after loading (as in Bernoulli–Euler theory), failure of the concrete is determined by the normal cross-sectional stresses, and the average normal strains along beam axis are equal in the bonded reinforcing longitudinal bars and surrounding concrete. These assumptions are also commonly used in the design of RC beams, for example, as described in the EN-1992-1-1:2004 standard. Under the mentioned conditions, the results shown in Figure 6 can be also applied to a damage estimation of individual fibers of RC beam. The authors used the numerical results in this way for damage level estimation of experimentally tested RC beams in point 5.

## 4. Determination of Times and Paths of the Fastest Sound Propagation Using Dijkstra’s Algorithm

**Figure 7:**
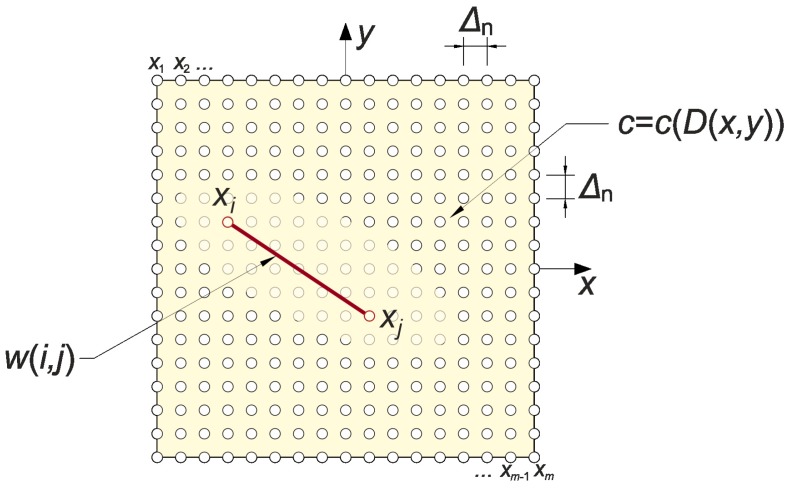
Illustrative orthogonal node arrangement for a graph in a plane problem (based on Reference [42]).

In this paper, Dijkstra’s algorithm [40] was used to evaluate the shape of paths of the fastest propagation of ultrasound waves and the time needed for them to travel in concrete elements. The physical basis for this type of calculations is Fermat’s principle [36]. Implementation of Dijkstra’s algorithm in this type of problem in a compact material area $V\in R^{2}$ (plane case) may be described as follows (e.g., Reference [42]). Let a sonic pulse be given at point S ∈ V which triggers the propagation of a specific wave type (e.g., longitudinal). Then, a graph from nodes with numbers *i* assigned to points $X_{i}\in V\;\left(i=1,2,\ldots ,m\right)$ should be built. *S* is one of the points $X_{i}$, and edges of the graph will be created only between those nodes which the $X_{i}X_{j}\subset V$ (i,j=1,2,…,m) condition is met for. Weight values w(i,j) for the particular edges will be the pass-through times of sound between the points, assuming that it is moving in a straight line section $X_{i}X_{j}$. Hence:

(21) $$w\left(i,j\right)=\int_{X_{i}}^{X_{j}}\frac{dl_{ij}}{c},\;$$

where: c—considered wave speed (m/s), $l_{ij}$—variable describing the position on a straight line section joining points $X_{i}$ and $X_{j}$ (m). In general, it can be seen that the more nodes evenly covering the V area (e.g., assuming an orthogonal grid of points $X_{i}$—Figure 7), the more accurate the calculation will be carried out. To increase the time efficiency of the method, when we are only interested in the time of sound propagation between selected two points, the number of nodes can be reduced only to those that lie within a reasonable distance from the straight line connecting these points and/or do not place nodes in a sub-areas where it is known that D≈0. If the graph is constructed, it is possible to use Dijkstra’s method in a standard way, i.e.,:

1. A set Q of all the nodes i=1,2,…,m is created and a vector d in which the times needed for a wave to travel from the node corresponding to the point $S=X_{1}$ to other nodes are recorded. First, it is assumed that $d_{1}=0$ and $d_{i}=\infty$ for i≠ 1.
2. In the set Q, a node (let us assign it a number j) is found for which the component d has a minimum value and is removed from this set. If the set Q is empty, the calculations are over.
3. In the case of other nodes i≠j belonging to the set Q, an inequality $d_{i}>d_{j}+w\;\left(i,j\right)$ is checked. If the inequality is satisfied, the value of $d_{j}+w\;\left(i,j\right)$ is assigned to the component $d_{i}$. The algorithm returns to point 2.

In the final effect of the algorithm operation, the vector components $d_{i}$ are equal to the time after which, from the moment of excitation of the wave at point *S*, it reaches the point $X_{i}$ assuming that the wave can only move on the edges of the graph. Remembering during the calculation also the next nodes indicated in point 3 of the algorithm, you can recreate the path of the fastest wave propagation in the graph.

### 4.1. Calculation Example 1

The primary purpose of the example is to show to what extent a change in stiffness in a localized, elastically degraded concrete zone can cause the paths of the fastest sound wave propagation to deflect. Another objective is to propose, on the basis of the performed analysis, a useful way to reduce the inaccuracies in tomographic imaging that may arise from this.

The example presents Dijkstra’s method of calculating the paths and times of the fastest longitudinal ultrasound wave propagation between the assumed transmitting/receiving points in the longitudinal section of the concrete beam model. One damaged zone was assumed to be formed in the beam in its entire cross-section of 0.4 m × 0.4 m (these dimensions were arbitrarily selected as typical, but this does not change the general conclusions which were finally drawn on this basis). The damaged area is located in the middle of a beam section of 1.2 m in length. It was assumed that the brittle damage changes the distribution of wave propagation velocity along its length in the x function according to the results shown in Figure 6b. It means that a damage level is uniform in a given cross-section of the beam. Consideration was limited to cases where a minimum ratio of $E_{D}/E_{0}$ in the damaged area was 0.9, 0.8, 0.6, or 0.2 with the development of the defect, and, due to the geometry of the task, imaging was performed on the section of the beam along its central vertical plane of symmetry. In order to simplify the calculations, the impact of any reinforcement on the results is not taken into account in the example. It should be obviously noted that this influence can be important for practical research and must not be ignored in the case of higher reinforcement ratios, in particular for the sound wave paths along the reinforcing bars. It is recommended to avoid such measurement situations as much as possible (e.g., Reference [1]). For this reason, it should also be emphasized that the conclusions resulting from the presented calculation examples can be used for quantitative analyses directly only for beams with a low reinforcement ratio and arrangement of propagation paths between the primary reinforcing bars and not along them and arms of stirrups, as well. In other situations, the impact of reinforcement should be taken into account—e.g., using appropriate correction factors, reducing the measured wave velocities so that they correspond to propagation conditions in non-reinforced concrete [1].

**Figure 8:**
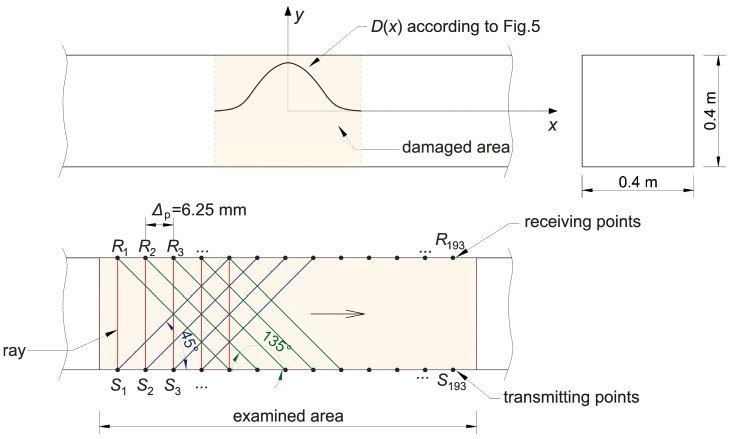
Scheme of the beam model with the assumed damage distribution and the system of transmitting/receiving points and rays.

The assumed scheme of the beam with transmitting/receiving points is shown in Figure 8. The transmitting/receiving points were selected in the system of opposite points distant from each other every $\Delta_{p}=6.25$ mm. Using Dijkstra’s algorithm, wave propagation times and paths were determined between the opposite points and with shifting of the receiving points relative to the transmitting points right or left so that the angle of inclination of the straight line between them to the axis x was 45° or 135°, respectively. In Figure 8, the tomographic rays corresponding to the wave propagation paths analyzed are also marked. Furthermore, in order to provide a convenient quantitative interpretation, the numerical results are presented in a dimensionless form, normalizing them to values that would have been obtained in the absence of the damage (i.e., in material characterized by longitudinal wave velocity equal to $c_{L0}$).

**Figure 9:**
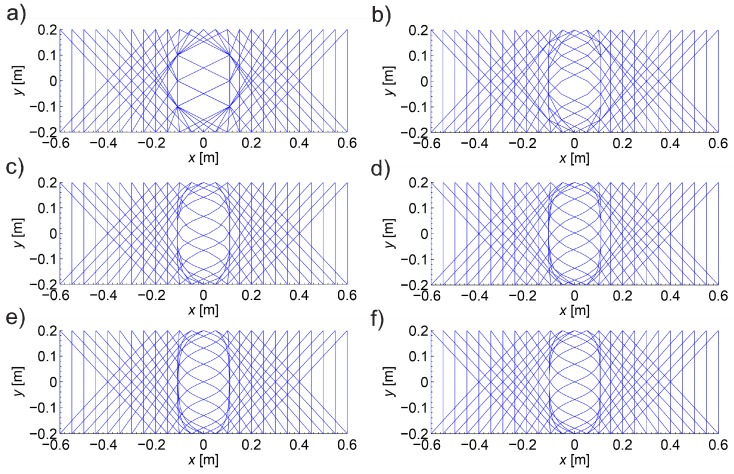
The shape of the paths of the fastest propagation between the selected transmitting/receiving points in the longitudinal section of the beam with the area damaged at $\min\left(E_{D}/E_{0}\right)=0.2$ and $\Delta_{n}\approx$ (**a**) 100 mm, (**b**) 50 mm, (**c**) 25 mm, (**d**) 12.5 mm, (**e**) 6.25 mm, and (**f**) 3.125 mm.

First, it was checked how the distance between the nodes of the graph ($\Delta_{n}$ according to Figure 7) in Dijkstra’s method influences the convergence of results. The graph nodes were placed in the transmitting/receiving points and inside the imaged longitudinal section in the intersection points of the orthogonal grid with the step of $\Delta_{n}$ vertically and horizontally. In order to reduce the calculation time, only the damaged area (in which $E_{D}/E_{0}\;$< 1) was covered by the internal nodes. The weights of the graph edges were determined from relation (21) by integrating the speed distributions shown in Figure 6b with a step of 1 mm using the rectangular method. Figure 9 shows paths of the fastest propagation illustratively only between transmitting/receiving points distant by 5 cm from each other, $\min\left(E_{D}/E_{0}\right)=0.2$ and $\Delta_{n}\approx 100$ mm, 50 mm, 25 mm, 12.5 mm, 6.25 mm, or 3.125 mm. Adoption of $\Delta_{n}$ in this way caused that the number of graph nodes in the width of the damaged area changed in extreme cases from 3 to 70. The width of this area can be read from the diagrams in Figure 5 and Figure 6. Table 1 shows the maximum relative changes of the determined wave propagation times on the fastest paths $t_{\mathrm{path},i}$ (s) and their lengths $L_{\mathrm{path},i}$ (m) as the step of the graph nodes decreases where i is the path index. When interpreting the shape of paths in Figure 9, it should be noted that if there is more than one path with the same wave propagation time due to the selection of points of graph nodes and symmetry of the problem geometry then the algorithm used shows the first one found among them. On the other hand, when analyzing the convergence of the solution (Table 1), it can be stated that, with the decrease in the step of the graph node grid, its relative changes become less and less significant and converge to zero. Due to the time needed to solve one such task and the owned computer hardware, further calculations were carried out using the solutions obtained at $\Delta_{n}=3.125$ mm (PC with processor 3.06 GHz and RAM 8 GB allowing to determine data for one path on average in approximately 4 min at $\Delta_{n}=3.125$ mm).

**Figure 10:**
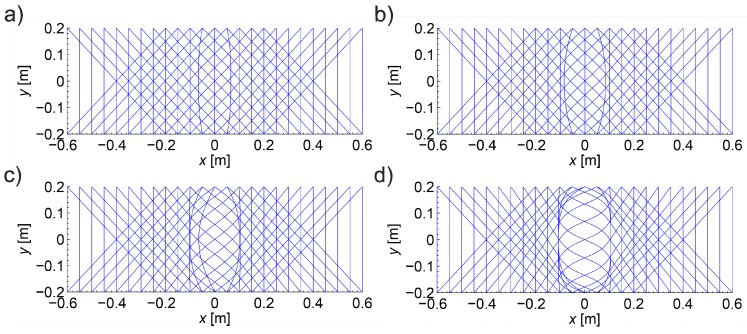
The shape of paths of the fastest propagation between the selected transmitting/receiving points in the longitudinal section of the beam depending on the degree of elastic degradation in the damaged zone $\min\left(E_{D}/E_{0}\right)=$ (**a**) 0.9, (**b**) 0.8, (**c**) 0.6, and (**d**) 0.2 (for $\Delta_{n}=3.125$ mm).

**Figure 11:**
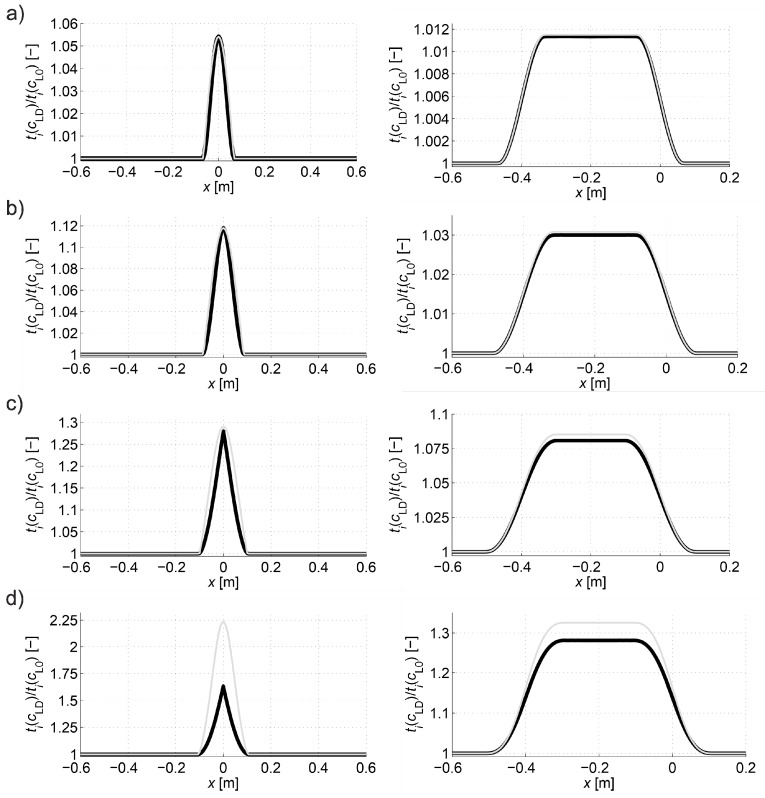
Times of wave propagation $t_{\mathrm{path},i}$ on the fastest paths, and $t_{\mathrm{ray},i}$ over the straight rays between the transmitting/receiving points in the different phases of the defect evolution: $\min\left(E_{D}/E_{0}\right)=$ (**a**) 0.9, (**b**) 0.8, (**c**) 0.6, and (**d**) 0.2. The results are presented as a function of the position of the transmitting points: a black line for the fastest paths determined by Dijkstra’s algorithm (for $\Delta_{n}=3.125$ mm); a grey line for the paths established as straight rays. The diagrams on the left refer to the paths connecting the opposite points and those on the right to the points lying diagonally at an angle of 45° to each other in relation to the axis x.

Figure 10 collectively shows the shape of selected paths of the fastest wave propagation depending on the degree of damage. It is evident that as the minimum ratio $E_{D}/E_{0}$ decreases the wave undergoes increasing deflection in the damaged area. At the same time, it is obvious that the time needed to travel such a path by the wave is shorter and shorter than the one that would be calculated with a simplistic assumption of its propagation in a straight ray. The scale of this difference is illustrated in Figure 11 showing how the propagation times change along the fastest paths and rays connecting consecutive opposite transmitting/receiving points and points lying diagonally at an angle of 45° to each other in relation to the axis of the beam. Figure 11 does not show the variability of times along the paths between points lying diagonally to each other at an angle of 135° relative to the axis x because it is the same as for those lying at an angle of 45° taking into account the symmetry. In order to make the diagrams more readable, they are also presented in the form of continuous curves. In the case of a damaged zone with a course perpendicular to the axis of the beam analysed in the example, it can be observed that greater differences in wave propagation times along the real paths and calculated on the assumption of their straightness occur for those that connect the opposite points and are increasing as the damage increases. These differences are much smaller for paths connecting the points diagonally. This observation may be used to reduce the inaccuracy of tomographic calculations due to the fact that $t_{\mathrm{path},i}$ (in fact, the times determined from measurements) are inserted in real situations in place of $t_{\mathrm{ray},i}$ in the system of Equation (3) where it is assumed in a simplistic way that $t_{\mathrm{ray}\;\mathrm{measured},i}$ = $t_{\mathrm{path},i}$. Therefore, in the case of propagation times only for opposite points, an approximate relationship can be proposed between $t_{\mathrm{path},i}$ and $t_{\mathrm{ray},i}$, i.e.,:

(22) $$t_{\mathrm{ray},i}\approx t_{\mathrm{ray}\;\mathrm{approx},i}=t_{\mathrm{path},i}+\beta \left(t_{\mathrm{path},i}-\min{\left(t_{\mathrm{path},i}\right)}_{i=1,2,\ldots ,I}\right),$$

where: $t_{\mathrm{ray}\;\mathrm{approx},i}$—approximated propagation times assuming a wave passage over the ray (s), β—non-negative factor [-].

Thus, the factor β scales propagation times from real paths by elongating them in relation to the minimum value in the direction of positive values so as to bring their course as close as possible to the times that would correspond to the passage of a wave over the straight ray. As a result, this will clearly result in a reduction of calculation errors. Analyzing the graphs shown in Figure 11, this factor can be determined, e.g., from the least squares method. However, in a situation of actual measurement, due to the lack of such data, this is impossible. Therefore, the authors propose to apply the following heuristic approach to the determination of β. Approximate solution of the system of Equation (3) can be alternatively obtained using Tikhonov’s method [51,52] minimizing the function of the mean square error of this solution (the so-called residual term $F_{\mathrm{res}}$) with additional consideration of the regularisation term $F_{\mathrm{reg}}$ (e.g., Reference [24]). Using this approach in the measurement situation, when in Equation (3) we replace the appropriate components in the vector b $t_{\mathrm{ray}\;\mathrm{measured},i}=t_{\mathrm{path},i}$ across $t_{\mathrm{ray}\;\mathrm{measured},i}=t_{\mathrm{ray}\;\mathrm{appox},i}\left(\beta \right)$, we will obtain:

(23) $$F_{\mathrm{res}}=\;\frac{1}{2}{\left\|Ax_{\alpha ,\beta }-b\left(\beta \right)\right\|}^{2},\;F_{\mathrm{reg}}=\frac{1}{2}\alpha {\left\|R(x_{\alpha ,\beta }-x_{0})\right\|}^{2}\;F_{\mathrm{res}}\left(x_{\alpha ,\beta }\right)+F_{\mathrm{reg}}\left(x_{\alpha ,\beta }\right)=\min\to x_{\alpha ,\beta }={\left(A^{T}A+\alpha R^{T}R\right)}^{-1}\left(A^{T}b\left(\beta \right)+\alpha R^{T}Rx_{0}\right),$$

where: α—regularization parameter [-], R—regularization matrix (m), $x_{\alpha ,\beta }$—solution of the problem for given values α and β (s/m), $x_{0}$—the point in the vicinity of which a regularized solution is sought (s/m). Matrix R was assumed in further consideration as a unitary one multiplied by 1 m for the sake of the unit account, and $x_{0}$ as the vector according to Equation (6). In the proposed method of determination of β, it is assumed that the less data contained in the vector b will be affected by measurement errors due to the selection of β, the smaller the values of the term $F_{\mathrm{reg}}$ in the case of an optimal solution to the problem. It is, therefore, proposed to determine the optimal value of the coefficient $\beta =\beta_{\mathrm{opt}}$ from the relationships:

(24) $$\beta_{\mathrm{opt}}=\max\;\left(g\left(z\right)\right)\;\mathrm{for}\;z\geq 0,$$

where

(25) $$g\left(z\right)=\arg\;\min\;\left(F_{\mathrm{reg}}\left(x_{\alpha =z,\beta >0}\right)\right)\;\mathrm{for}\;z\geq 0.$$

**Figure 12:**
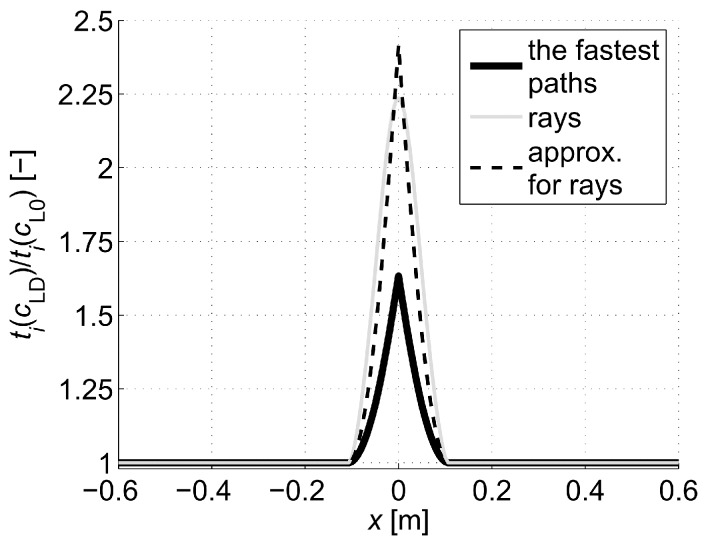
Propagation times $t_{\mathrm{path},i}$, $t_{\mathrm{ray},i}$ and $t_{\mathrm{ray}\;\mathrm{approx},i}$ between the opposite transmitting/receiving points for a defect with $\min\left(E_{D}/E_{0}\right)=0.2$. $t_{\mathrm{ray}\;\mathrm{approx},i}$ were calculated according to relation (22) with $\beta =\beta_{\mathrm{opt}}$.

Selection of $\beta_{\mathrm{opt}}$ as the maximum value of function (25), in turn, has the following meaning: determine what is the maximum possible increase in the propagation time of the wave in the vector b using formula (22) with a minimum effect of regularization so that the original information contained in system of Equation (3) is lost as little as possible at the same time. Using formulas (22)–(25), the following values of $\beta_{\mathrm{opt}}$ were obtained as in Table 2 for the data from the graphs in Figure 11. Illustratively, the variability of the wave propagation time $t_{\mathrm{ray}\;\mathrm{approx},i}$ modified according to relation (22) between opposite transmitting/receiving points at $\beta =\beta_{\mathrm{opt}}$ and $\min\left(E_{D}/E_{0}\right)=0.2$ is shown in Figure 12. At the same time, it was compared with $t_{\mathrm{ray},\;i}$ and $t_{\mathrm{path},i}$, where $t_{\mathrm{ray}\;\mathrm{approx},i}$ has a much more similar course to that of $t_{\mathrm{ray},i}$ than $t_{\mathrm{path},i}$.

**Figure 13:**
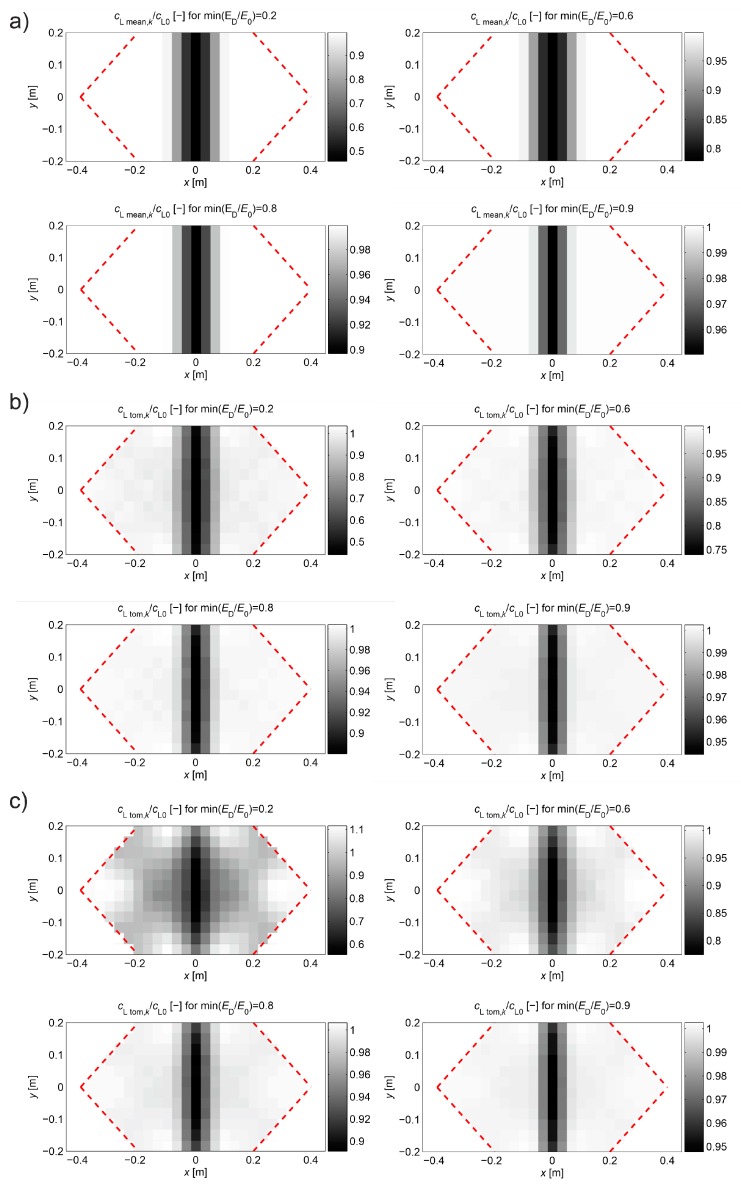
Originally assumed distributions of wave velocity in the longitudinal section of the beam in different stages of defect evolution (**a**) and their tomographic reconstructions according to Equation (3): (**b**) calculated using the propagation times $t_{\mathrm{ray},i}=t_{\mathrm{ray}\;\mathrm{approx},i}$ for the paths connecting the opposite points and $t_{\mathrm{ray},i}=t_{\mathrm{path},i}$ for the paths connecting the points diagonally, (**c**) calculated using only the propagation times $t_{\mathrm{ray},i}=t_{\mathrm{path},i}$ (red dotted lines—explanation in the text).

In order to assess the correctness of the proposed approximation method, appropriate tomographic reconstructions of the longitudinal wave velocity maps in the beam model section from Figure 8 are presented in Figure 13. The reconstructions were determined by the randomized Kaczmarz method in accordance with the information presented in point 2, where $c_{L0}$ was adopted as $c_{L\;\mathrm{ref}}$. The results are shown only for the central area of the beam separated by a red dashed line through which all types of rays passed due to their inclination. Wave velocity distributions in the damaged area according to Figure 6b were assumed with minimal ratio $E_{D}/E_{0}$ equal to 0.9, 0.8, 0.6, or 0.2. The resolution of $\delta_{1}\times \delta_{2}=3.33$ cm × 3.33 cm was applied, as well as the number and arrangement of rays, as shown in Figure 8. For comparison purposes, in addition to the reconstruction with the use of times $t_{\mathrm{ray}\;\mathrm{approx},i}$ according to relation (22) and $t_{\mathrm{path},i}$, the original map is also shown on the basis of data from Figure 6b, but in the resolution used, i.e., in each cell, its average speed value is taken from the formula:

(26) $$c_{L\;\mathrm{mean},k}=\frac{\int_{A_{k}}\;c_{L}\;dx\;dy}{A_{k}},$$

where: $A_{k}$—rectangular area occupied by the k-th cell (m^2^). Integral (26) was calculated in an approximate way using the rectangular method, taking the step of integration after x and y, respectively as $\delta_{1}/10$ and $\delta_{2}/10$. Therefore, relative errors were calculated for each reconstruction: global—mean square $e_{g}=\surd (\sum_{k=1}^{K}(c_{L\;\mathrm{mean},k}-c_{L\;\mathrm{tom},k}{)}^{2}/\sum_{k=1}^{K}c_{L\;\mathrm{mean},k}^{2})$ and local—maximal $e_{l\;\max}=\max((c_{L\;\mathrm{mean},k}-c_{L\;\mathrm{tom},k})/c_{L\;\mathrm{mean},k}{)}_{k=1,2,\ldots ,K}$, where $c_{L\;\mathrm{tom},k}$ was determined from system of Equation (3). Because $c_{L\;\mathrm{mean},k}$ is not known in a real measuring situation, it is also necessary to introduce a measure which will allow to estimate the reconstruction errors due to the assumption of straight-lined rays without this information.

In this paper, it is proposed to calculate index $d_{\mathrm{cL}\;\mathrm{ray}\;\max}$: the maximal relative difference between $c_{L\;\mathrm{ray}\;\mathrm{measured},i}$ (wave velocities averaged over the i-th ray based in real directly on the measured propagation times) and $c_{L\;\mathrm{ray}\;\mathrm{approx},i}$ (wave velocities averaged over the i-th ray calculated, respectively, on the basis of approximated propagation times according to relation (22)). This leads to the formula:

(27) $$d_{\mathrm{cL}\;\mathrm{ray}\;\max\;}=\max{\left(\frac{c_{L\;\mathrm{ray}\;\mathrm{measured},i}-c_{L\;\mathrm{ray}\;\mathrm{approx},i}}{c_{L\;\mathrm{ray}\;\mathrm{measured},i}}\right)}_{i=1,2,\ldots ,I}=\max{\left(1-\frac{t_{\mathrm{path},i}}{t_{\mathrm{ray}\;\mathrm{approx},i}}\right)}_{i=1,2,\ldots ,I}\;.$$

A summary of $e_{g}$, $e_{l\;\max}$, and $d_{\mathrm{cL}\;\mathrm{ray}\;\max\;}$ is presented in Table 3, depending on the adopted calculation strategy, while for $d_{\mathrm{cL}\;\mathrm{ray}\;\max\;}$, the exact value of it is given in a comparative manner (if calculated with the use of times of propagation $t_{\mathrm{ray},i}$ instead of $t_{\mathrm{ray}\;\mathrm{approx},i}$).

The analysis of the presented results shows that the reconstruction error increases with the degree of development of localized brittle damage. If propagation times $t_{\mathrm{path},i}$ are used in vector b of Equation (3) (as would be obtained directly from the measurement in the real situation), then, in the analyzed case, the relative decrease of Young’s modulus in the damaged zone from 10% to 80% is connected with the increase of $e_{g}$ from approximately 0.002 to 0.09. In parallel, $e_{l\;\max}$ changes from 0.01 to 0.70. Introduction to the vector b propagation times for the opposite transmitting/receiving points according to formula (22) with the optimum value of β allows reducing the relative reconstruction error of both $e_{g}$, as well as $e_{l\;\max}$, for ranges from approximately 0.001 to 0.03 and from 0.007 to 0.19, respectively. The use of formula (22) also allows for a more accurate evaluation of the shape and spatial range of the defect (Figure 13), i.e., regardless of the degree of damage, its original shape is visualized correctly. Using only propagation times $t_{\mathrm{path},i}$ in the calculations allows to strictly assess the shape of the analyzed defect only when Young’s modulus drops to about 20%. On the other hand, the value of $d_{\mathrm{cL}\;\mathrm{ray}\;\max}$ also allows a rough estimation of the differences in the reconstructed wave velocity maps that may occur due to the adoption of the fastest straight-line propagation paths in calculations (although, taking into account the limitations that result from its definition according to Equation (27)). Its advantage in turn is that it can be determined in research only on the basis of measurement data. It starts to increase noticeably when Young’s modulus drops in the defected zone above 20%.

### 4.2. Calculation Example 2

**Figure 14:**
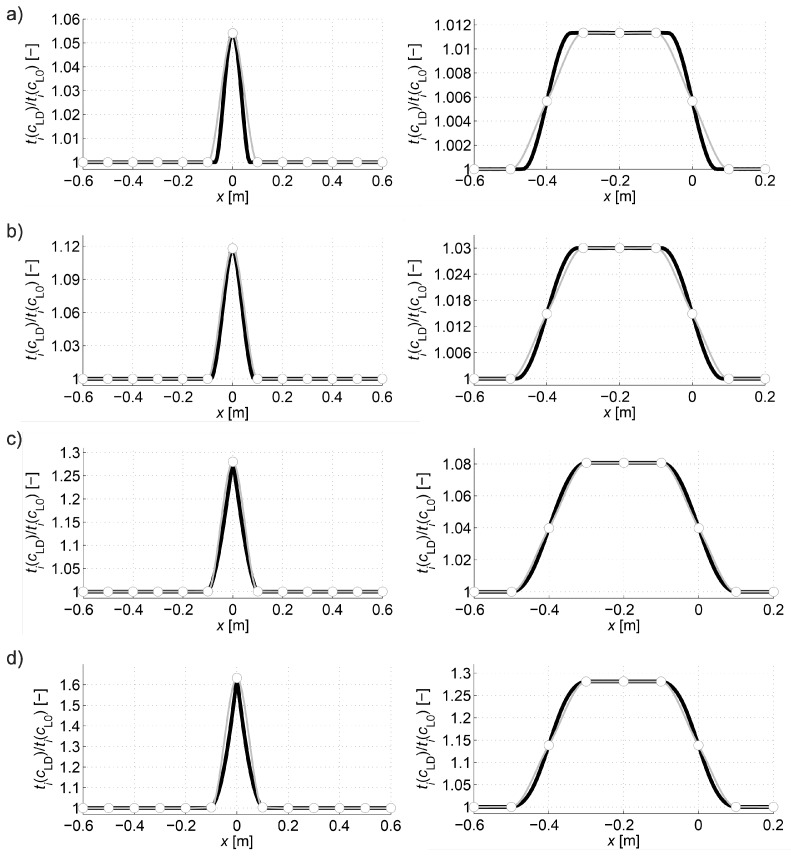
Times of wave propagation $t_{\mathrm{path},i}$ and $t_{\mathrm{path}\;\mathrm{int},i}$ on the fastest paths between transmitting/receiving points in different phases of defect evolution: $\min\left(E_{D}/E_{0}\right)=$ (**a**) 0.9, (**b**) 0.8, (**c**) 0.6, and (**d**) 0.2. The results are presented as a function of the position of the transmitting points: a black line for the fastest paths determined by Dijkstra’s algorithm (for $\Delta_{n}=3.125$ mm); a grey line in case of interpolation with nodes marked with circles. The diagrams on the left refer to the paths connecting the opposite points and those on the right to the points lying diagonally at an angle of 45° to each other in relation to the axis x.

The purpose of the second calculation example is to show that the desired resolution of the image of changes in ultrasound wave velocity around localized damage can be obtained by using a reduced number of transmitting/receiving points. To this end, so-called fictitious transmitting/receiving points should be introduced for which propagation times will be interpolated on the basis of data from the original set of points (e.g., Reference [33,47]). In a real measurement situation, this may often result in a significant reduction of transmitting/receiving points, costs and time consumption of tests. The simulation of such a measurement strategy for the data of the first calculation example is shown below. The same geometry of the beam model and the method of its damage as in the first example were adopted, but the number of points for which the original information about wave propagation times is available was reduced in such a way that the gaps between them amount to $\Delta_{p}=10$ cm. There are fictitious points every 6.25 mm between them at the same intervals as the points in the first example. For each group of paths, depending on the location of their transmitting/receiving points relative to each other (the opposite points, the points lying diagonally at an angle of 45° and 135° in relation to the axis x) times for paths connecting fictitious points were interpolated. The interpolation in the function of transmitting points position was carried out with the use of a cubic Hermite spline assuming continuity to the first derivative (*pchip* function in the MATLAB program environment was used). The results are shown in Figure 14, where interpolation nodes were marked with circles. They were also compared with the propagation times calculated with Dijkstra’s algorithm in the first example. Figure 14 does not show the variability of times for the paths between the points lying diagonally to each other at an angle of 135° relative to the axis x because it is the same as for those lying at an angle of 45° taking into account the symmetry of the problem. In order to make the graphs easier to read, the interpolated times and those of the first example are presented in the form of continuous curves. Relative global interpolation errors are summarized in Table 4, i.e., $e_{g}=\surd (\;\sum_{i=1}^{I}(\;t_{\mathrm{path},i}-t_{\mathrm{path}\;\mathrm{int},i}{\;)}^{2}/\sum_{i=1}^{I}t_{\mathrm{path},i}^{2})$ was calculated where $t_{\mathrm{path}\;\mathrm{int},i}$ (s) is the interpolated propagation time of the wave on the i-th path.

In the same way as in the first example, the propagation times for the fastest ultrasound wave paths connecting opposite transmitting/receiving points were modified to bring them as close as possible to the propagation times of straight rays, but with the use of times $t_{\mathrm{path}\;\mathrm{int},i}$, i.e.,:

(28) $$t_{\mathrm{ray},i}\approx t_{\mathrm{ray}\;\mathrm{approx},i}=t_{\mathrm{path}\;\mathrm{int},i}+\beta \left(t_{\mathrm{path}\;\mathrm{int},i}-\min{\left(t_{\mathrm{path}\;\mathrm{int},i}\right)}_{i=1,2,\ldots ,I}\right).$$

**Figure 15:**
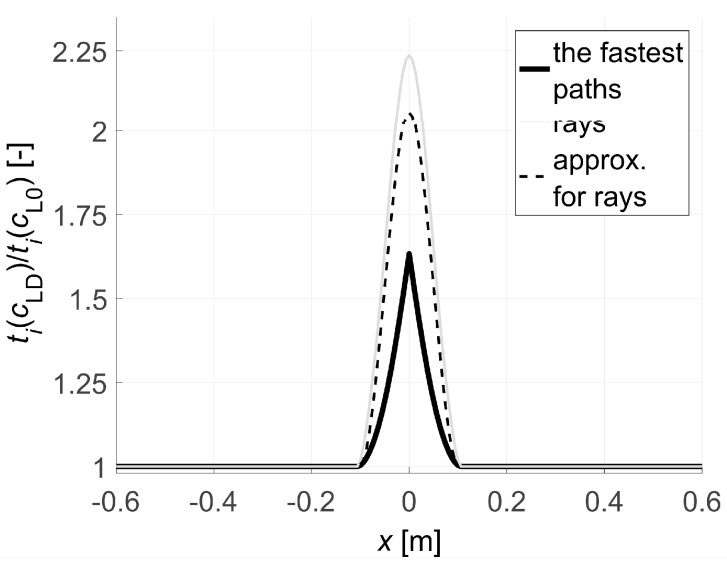
Propagation times $t_{\mathrm{path},i}$, $t_{\mathrm{ray},i}$ and $t_{\mathrm{ray}\;\mathrm{approx},i}$ between the opposite transmitting/receiving points for a defect with $\min\left(E_{D}/E_{0}\right)=0.2$. $t_{\mathrm{ray}\;\mathrm{approx},i}$ were calculated according to relation (28) with $\beta =\beta_{\mathrm{opt}}$.

Then, using formulas (23)–(25), the values of $\beta_{\mathrm{opt}}$ were obtained for data from Figure 14 as in Table 5. Illustratively, the variability of the wave propagation time $t_{\mathrm{ray}\;\mathrm{approx},i}$ modified according to relation (28) between opposite transmitting/receiving points at $\beta =\beta_{\mathrm{opt}}$ and $\min\left(E_{D}/E_{0}\right)=0.2$ is shown in Figure 15. At the same time, it was compared with $t_{\mathrm{ray},i}$ and $t_{\mathrm{path},i}$, where $t_{\mathrm{ray}\;\mathrm{approx},i}$ has also a much more similar course to that of $t_{\mathrm{ray},i}$ than $t_{\mathrm{path},i}$.

**Figure 16:**
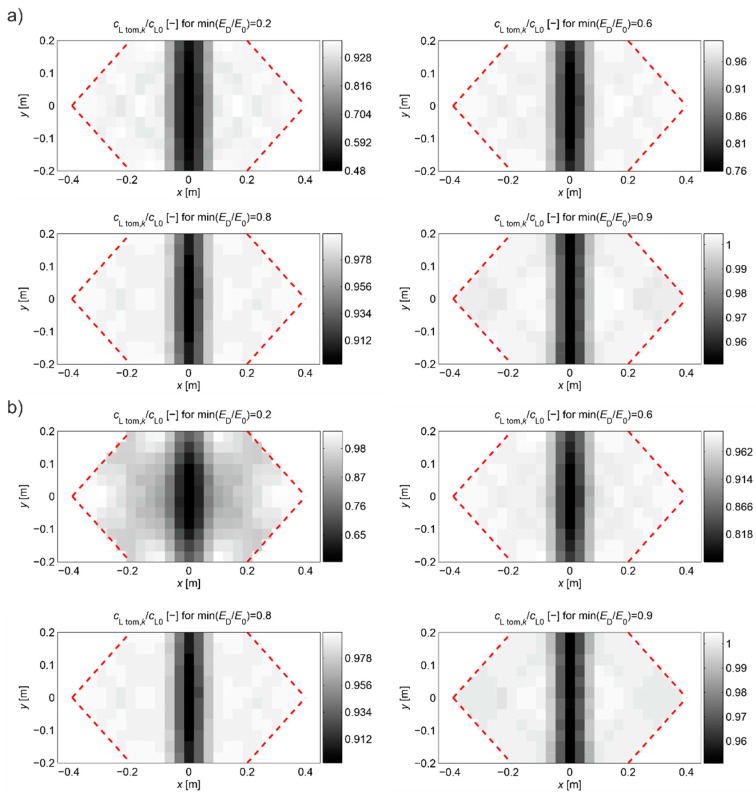
Tomographic reconstructions of the wave velocity distribution from Figure 13a according to Equation (3): (**a**) calculated with the propagation times $t_{\mathrm{ray},i}=t_{\mathrm{ray}\;\mathrm{approx},i}$ for the paths connecting the opposite points and $t_{\mathrm{ray},i}=t_{\mathrm{path}\;\mathrm{int},i}$ for the paths connecting the points diagonally, (**b**) calculated only with the propagation times $t_{\mathrm{ray},i}=t_{\mathrm{path}\;\mathrm{int},i}$ (red dotted lines—explanation in the text).

In order to assess the correctness of the proposed propagation time interpolation method, tomographic reconstructions of the wave velocity maps in the beam model longitudinal section from Figure 8 are presented in Figure 16. The reconstructions were determined by the randomized Kaczmarz method in accordance with the information presented in point 2 where $c_{L0}$ was adopted as $c_{L\;\mathrm{ref}}$. The results are shown only for the central area of the beam section separated by a red dashed line through which all types of rays passed due to their inclination. For comparison purposes, reconstructions with the use of times $t_{\mathrm{ray}\;\mathrm{approx},i}$ according to relation (28) and $t_{\mathrm{path}\;\mathrm{int},i}$ were presented. For the individual reconstructions, their relative errors were also calculated: global—mean square $e_{g}$, local—maximal $e_{l\;\max}$. The relative maximal difference in average speed over rays $d_{\mathrm{cL}\;\mathrm{ray}\;\max\;}$ was also determined due to the use of $t_{\mathrm{ray},i}=t_{\mathrm{ray}\;\mathrm{approx},i}$ or $t_{\mathrm{ray},i}=t_{\mathrm{path}\;\mathrm{int},i}$, i.e., in this particular case:

(29) $$d_{\mathrm{cL}\;\mathrm{ray}\;\max}=\max{\left(1-\frac{t_{\mathrm{path}\;\mathrm{int},i}}{t_{\mathrm{ray}\;\mathrm{approx},i}}\right)}_{i=1,2,\ldots ,I}.$$

A summary of $e_{g}$, $e_{l\;\max}$ and $d_{\mathrm{cL}\;\mathrm{ray}\;\max\;}$ is presented in Table 6, depending on the calculation strategy adopted, where the exact value of $d_{\mathrm{cL}\;\mathrm{ray}\;\max\;}$ is also shown for a comparison as in Table 3.

Analyzing the presented results one can draw general conclusions similarly to the reconstructions from the first example. In addition, it can be noted that reconstruction errors, compared to those in Table 3 for damaged zones with Young’s modulus drop of more than 10%, were decreased by up to 2 times at most. Again, it can also be stated that, in the case of a defect in a concrete member with a higher degree of elastic degradation, the introduction into the vector b in Equation (3) of wave propagation times after appropriate scaling (Equation (28) with the optimal value of β) allows for effective, even several fold reduction of calculation errors and more correct evaluation of the defect shape. The obtained results also confirm that the use of so-called fictitious points with interpolated propagation times allows to increase the resolution of tomographic reconstructions of elastically degraded concrete areas without the need to use “too dense” system of real transmitting/receiving points. In this case, the value of $d_{\mathrm{cL}\;\mathrm{ray}\;\max}$ also allows rough estimation of differences in reconstructed wave velocity maps, which can occur due to the adoption of the fastest propagation paths as straight in calculations. However, the use of fictitious points causes that it deviates from the exact values much more than in the first example. Nevertheless, it is important at this point that it starts to increase noticeably when Young’s modulus in the defect drops above 20%, and, for example, such an estimation can be rationally increased by 2–3 times for safety reasons.

## 5. Experimental Study

As part of the tomographic experiments, studies were carried out on RC elements after cracking initiation—three beams on a laboratory scale and one prefabricated beam on a natural scale. Since the calculation methodology presented in point 3 concerned the detection of elastically degraded zones with a course perpendicular to the beam axis, the experiments illustrating the possibilities of its practical application also focused on the evaluation of this type of defects. Hence, the laboratory beams were bent until the first perpendicular cracks were formed in the middle area. In the second case, a prefabricated industrially manufactured beam, that was damaged during transport to the construction site, was inspected. There were cracks perpendicular to the beam axis and visible to the naked eye. This beam was specially selected for an assessment to also test the presented calculation method in near-real conditions. An important aspect of this study was also the willingness to check whether ultrasound tomography in the presented approach could potentially be used for quality control of prefabricated RC elements in industrial conditions.

The cross-sections of beams for tomographic imaging were selected so that the measurements were disturbed as little as possible by their reinforcement (between longitudinal bars and vertical arms of stirrups) [1,53]. In the case of laboratory beams, it was also decided to show how changing the care method can affect tomographic detection of brittle damage. For this purpose, three samples were stored under water for 1 to 28 days from the time of forming. Two of them were tested after 28 days and the third one after 35 days (after its removal from water at the age of 28 days and storage at the room conditions for the next 7 days). The storage temperature of all the beams was about 20 °C. Taking into account also disturbances which may be caused by uneven distribution of humidity [53], the beams were examined in conditions in which the distribution of humidity in them would be as homogeneous as possible. The 28-day laboratory beams were tested for up to about 1 h after the removal from the water bath, and the 35-day beam, after the removal from the water, was protected by polyethylene film against moisture exchange with the ambient air until the test. In turn, the prefabricated beam was stored about 7 months before the test inside the laboratory at an average relative humidity of about 50% and a temperature of 20 °C. The age of the prefabricated beam at the time of testing was about 9 months.

**Figure 17:**
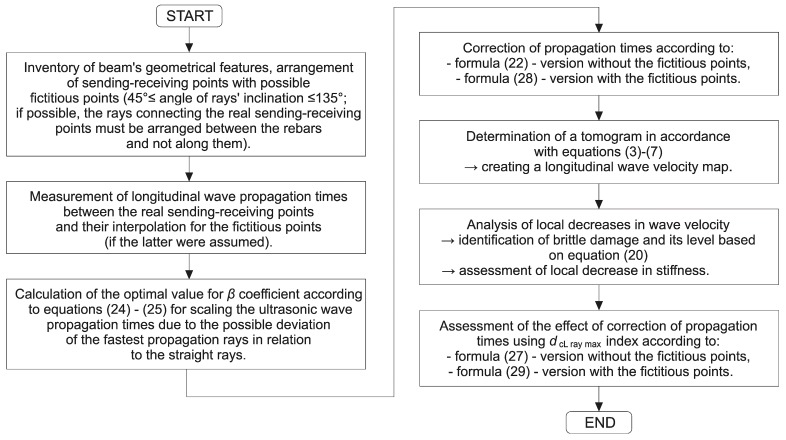
Flowchart of the calculation process.

On the basis of theoretical considerations discussed in previous points, a detailed course of tomographic measurements and method of processing data obtained this way was also established and used during own experiments. The general scheme of this procedure is shown in Figure 17.

### 5.1. Laboratory Beams

**Figure 18:**
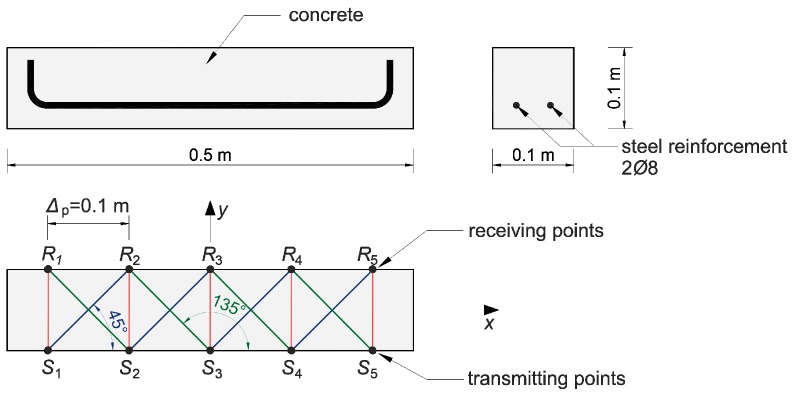
Scheme of the beams and the assumed system of transmitting/receiving points and rays.

**Figure 19:**
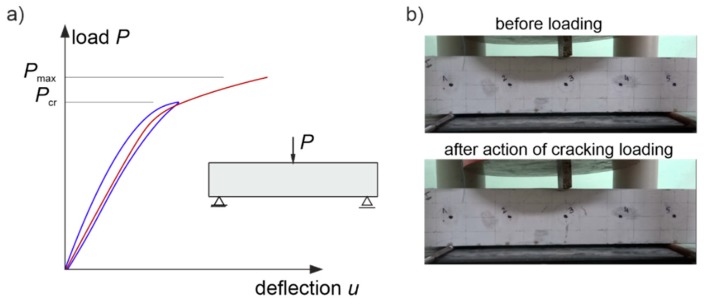
(**a**) Beam loading scheme. (**b**) Illustrative pictures of beam No. 3 before and after an action of cracking load.

A diagram of the beams is shown in Figure 18. Their dimensions were 10 cm × 10 cm × 50 cm and they were made of ready market mixture of concrete with a mean compression strength of $f_{\mathrm{cm},\mathrm{cube}}=48$ MPa after 28 days. The maximum aggregate diameter was $d_{a\;\max}=8$ mm. The longitudinal lower reinforcement consisted of two bars of 8 mm in diameter, made of steel with characteristic yield point declared by the manufacturer of $f_{\mathrm{yk}}=400$ MPa. The studies consisted in the measurement of the time of propagation of the longitudinal ultrasound wave using a Pundit-Lab tester and transreceiver heads with a frequency of 250 kHz. The coupling of the heads and element was provided by special gel for ultrasonic tests. The adopted system of transmitting/receiving points distant from each other by $\Delta_{p}=10$ cm is shown in Figure 18. Tomographic rays were assumed between the opposite points and lying diagonally at an angle of 45° and 135° in relation to the beam axis (Figure 18). The longitudinal vertical section of the beams for tomographic imaging was located in the middle between the reinforcement bars. For comparison, the ultrasound tests were carried out in two stages: before and after the first load-unload cycle in static three-point bending (Figure 19). The first load stage was finished at the moment when the first cracks appeared, controlling the registered load (P) and deflections at the centre of the span (u), i.e., until the slope change occurs in the function P−u. After the second stage of ultrasonic testing, the beams were bent until their load capacity was exhausted. The obtained values of the cracking ($P_{\mathrm{cr}}$) and maximum ($P_{\max}$) loads are summarized in Table 7.

Taking into account the measured longitudinal wave propagation times and basic frequency of ultrasonic pulses, the average wavelengths for beam No. 1, 2, and 3 were, respectively, ~1.7 cm, ~2.0 cm, and ~2.1 cm before the loading and ~1.7 cm, ~1.8 cm, and ~2.0 cm after the loading. At the same time, it allowed satisfying the basic requirement described in ASTM D2845-08 regarding the selection of frequency so that measurable longitudinal ultrasonic waves could be generated in the samples, i.e.,: dominant wavelength ≥ 3× the average grain size equal to ~4 mm.

**Figure 20:**
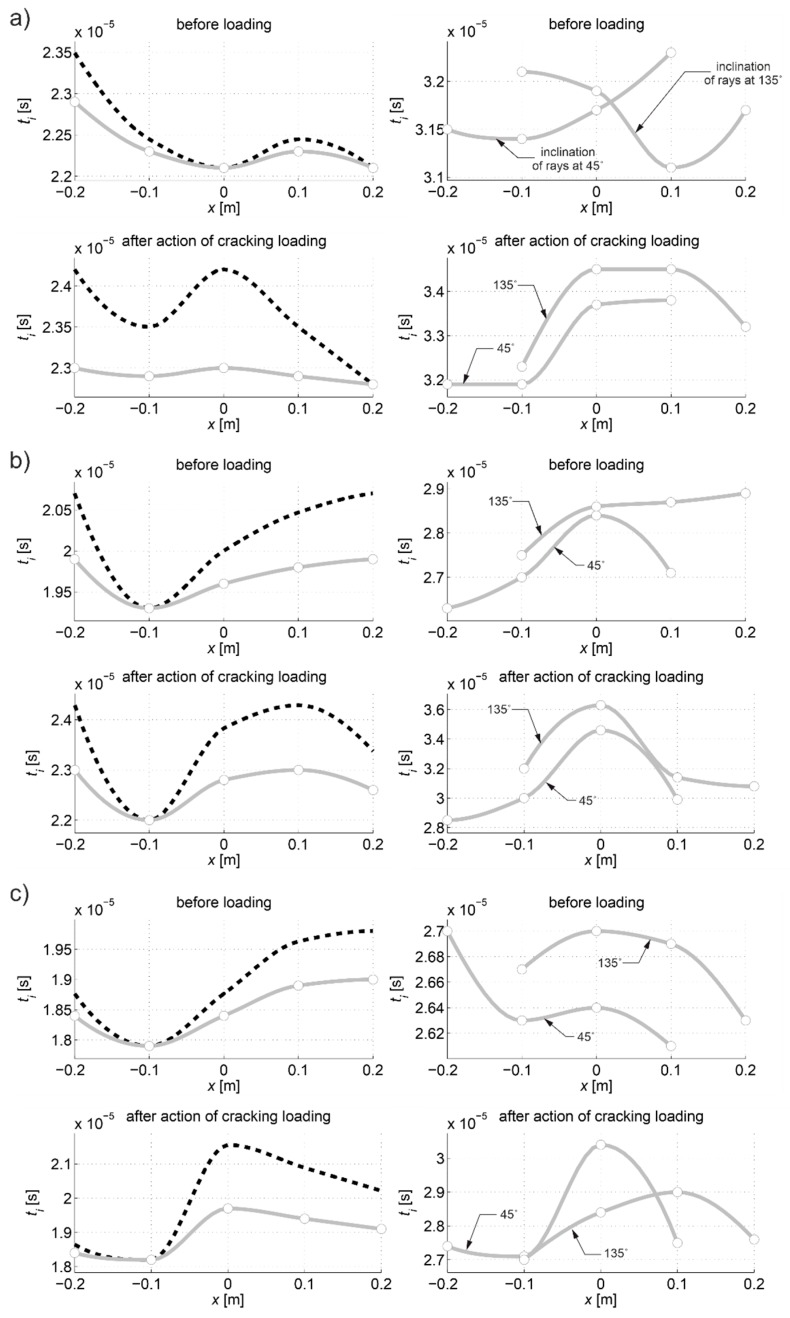
Propagation times $t_{\mathrm{path}\;\mathrm{int},i}$ and $t_{\mathrm{ray}\;\mathrm{approx},i}$ between the transmitting/receiving points in the beams before and after an action of cracking load: (**a**) No. 1, (**b**) No. 2, and (**c**) No. 3. The results are presented as a function of the position of the transmitting points: $t_{\mathrm{path}\;\mathrm{int},i}$ a grey line with nodes marked with circles, and $t_{\mathrm{ray}\;\mathrm{approx},i}$ a black, dashed line. The diagrams on the left refer to the paths connecting the opposite points and those on the right to the points lying diagonally at an angle of 45° and 135° to each other in relation to the axis x.

**Figure 21:**
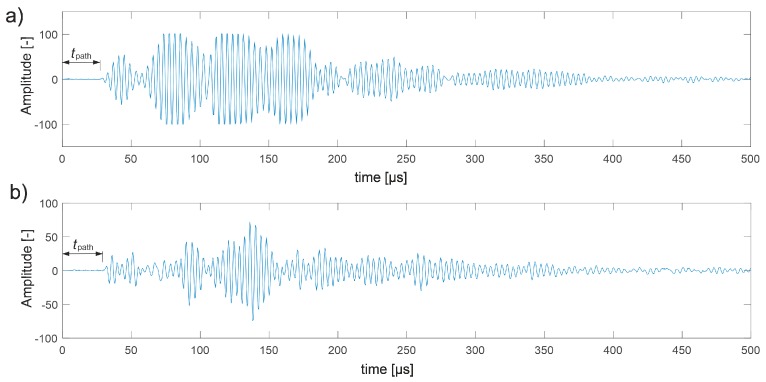
Example of signal recorded by the receiving head (transmitting point No. 4 and receiving point No. 3 in beam No. 3): (**a**) before load; (**b**) after an action of cracking load.

The first visible crack appeared in each case in the middle of the beam span as perpendicular to the beam axis (Figure 19). Measured and interpolated longitudinal wave propagation times $t_{\mathrm{path}\;\mathrm{int},i}$ are shown in Figure 20 where interpolated times are determined using a cubic Hermite spline. The diagrams also show $t_{\mathrm{ray}\;\mathrm{approx},i}$ determined according to relation (28) with $\beta =\beta_{\mathrm{opt}}$ according to (24), (25). In Figure 20, by comparing wave propagation times before and after the loading, the evolution of cracks can be clearly observed and their location initially made in the sections where these times have increased the most. It can also be seen that not taking into account the increase in the propagation time of ultrasonic pulses along the straight rays compared to the times measured for the fastest paths would lead to their underestimation of approximately 5–8% in the most damaged areas of the beams. Figure 21 shows an example of a recorded signal by the receiving head together with a reading the time of longitudinal wave propagation. In Figure 21, the signal caused by longitudinal wave propagation is visible first, and, a moment later, the signal connected with the propagation of transverse and Rayleigh waves of much higher amplitude can be noticed, but without the possibility of precise distinction of the initial moment of their registration. This was also the main reason why the authors decided to use longitudinal waves in their studies, taking into account the capabilities of their research equipment.

**Figure 22:**
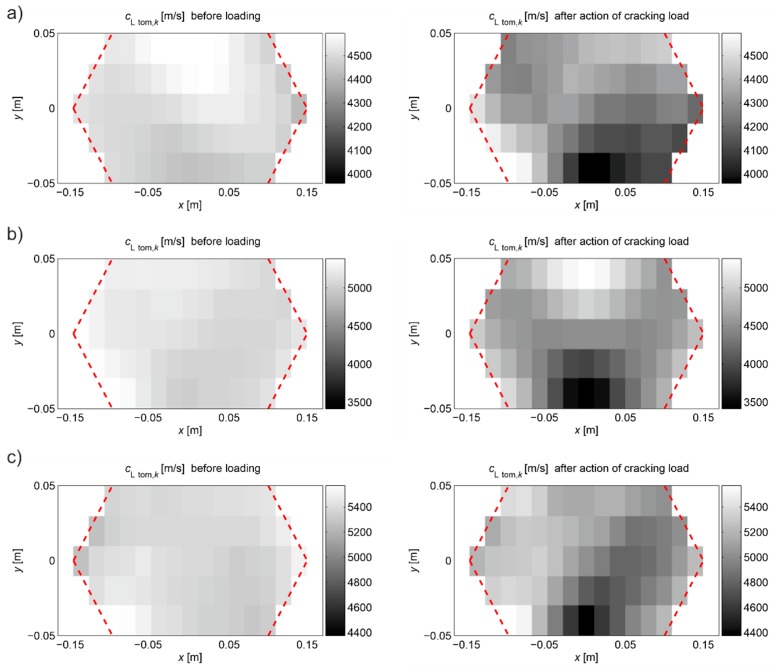
Tomographic reconstructions of the distribution of wave velocity according to Equation (3) in the longitudinal section of the beam before and after an action of the cracking load: (**a**) No. 1, (**b**) No. 2, and (**c**) No. 3 (red dotted lines—explanation in the text).

Figure 22 shows the tomographic reconstructions of longitudinal wave velocity maps in the vertical longitudinal section of the beams. Reconstructions were determined by a randomized Kaczmarz method in accordance with the information presented in point 2, where for $c_{L\;\mathrm{ref}}$ the maximum measured mean speed of the longitudinal wave over all rays before loading was adopted (4547 m/s, 5377 m/s, and 5587 m/s for beams No. 1, No. 2, and No. 3, respectively). The resolution of $\delta_{1}\times \delta_{2}=2$ cm × 2 cm was applied and the arrangement of rays as in Figure 18 with addition of rays between real rays connecting fictitious transmitting/receiving points at a distance of every 1 cm. The results are shown only in the middle area of the beam section, separated by a red dashed line through which all types of rays passed due to their inclination. The maps presented here are calculated on the basis of Equation (3) with propagation times $t_{\mathrm{ray},i}=t_{\mathrm{ray}\;\mathrm{approx},i}$ in accordance with (28) for the paths connecting the opposite points and $t_{\mathrm{ray},i}=t_{\mathrm{path}\;\mathrm{int},i}$ for the diagonal paths. For this purpose, the values of $t_{\mathrm{path}\;\mathrm{int},i}$ are taken as shown in Figure 20. Optimal coefficients β necessary for the determination of $t_{\mathrm{ray}\;\mathrm{approx},i}$ were calculated in accordance with (24) and (25) and their values are summarized in Table 8. The table also shows the values of $d_{\mathrm{cL}\;\mathrm{ray}\;\max}$ determined from Equation (29).

Figure 22 shows clearly formed elastically degraded zones caused by load $P=P_{\mathrm{cr}}$. Because of the static scheme of the bent beams, they were created in the middle of their span in the lower part of the cross-section. Based on Equation (20), the maximal tangential changes of Young’s moduli, defined by the ratio of $\min\left(E_{D}/E_{0}\right)$ at the level 0.81, 0.50, and 0.68 in beam No. 1, No. 2, and No. 3, respectively, can be estimated for these zones. In turn, their widths in these places is within the range of 16–18 cm. They are very close to the width of the localized elastically degraded zones which were calculated theoretically in point 3. For the ratio $\min\left(E_{D}/E_{0}\right)$ in the 0.5–0.8 interval, this corresponds to the width of the damaged area from approximately 17 cm to 19 cm, which can be read from Figure 5a or Figure 6a. This result indirectly pre-confirms the validity of the identification method for the internal length $l_{c}$ of concrete proposed in point 3. However, these preliminary out-comes need necessarily further intense experimental verification. In addition, it can be seen in Figure 22 that, under real conditions, the heterogeneity of the concrete itself can have a non-negligible effect on the results, as can be seen in the reconstructions of wave velocity distributions before the loading the beams. This is also evidenced by the determined values of $d_{\mathrm{cL}\;\mathrm{ray}\;\max}$ at this stage of the study (Table 8). In turn, after the bending moment load initiating the appearance of the first cracks, the values of $d_{\mathrm{cL}\;\mathrm{ray}\;\max}$ from Table 8 show that, in the investigated case, not taking into account deflections of the fastest propagation paths may lead to overestimation of the velocity of longitudinal waves on average by approximately 5–9%.

Another interesting issue that can be seen is that, despite the same period of care in the water of all beams, the speed of longitudinal waves in the intact configuration in beam No. 1 (stored an additional 1 week in the room conditions and isolation) was lower by about 20% if compared to the speed in beams No. 2 and 3 (which were tested right after removing from water). The explanation for this may be the fact of the phenomenon of self-drying of young concrete as a result of hydration processes [54]. On the other hand, as predicted by poromechanics [55], the initial tangent Young’s modulus of the porous material and the Poisson’s ratio in the state of full saturation is higher than in the dry state, which may result in a corresponding decrease in longitudinal wave velocity. In the case of cement matrix materials, such changes in Young’s modulus and Poisson’s ratio were measured by means of static tests among others in works [56,57,58]. For example, the Young’s modulus of concrete at the age of 51 days with a mean compressive strength $f_{\mathrm{cm},\mathrm{cube}}=64.6$ MPa (in the state of water saturation) varied from 47.2 GPa to 45.1 GPa and the Poisson’s ratio from 0.25 to 0.15 at the transition from the saturation with water to a moisture concentration reduced by approximately 2.2% by weight [58]. Assuming proportional changes for dynamic values of this parameters and taking into account the formula expressing the speed of longitudinal waves:

(30) $$c_{L}=\sqrt{\frac{E_{0}\left(1-\nu \right)}{\rho \left(1-2\nu \right)\left(1+\nu \right)}}\;,$$

where: ν—Poisson’s ratio [-], ρ—density (kg/m^3^), its relative decrease with the quoted range of changes in $E_{0}$, ν and density would be about 8%. The effect can be further enhanced by micro-cracks in concrete arising as a result of its autogenic and/or moisture shrinkage [54] (the latter in the case of absence of drying protection). The comparison of preliminary results from beams No. 1, 2, and 3 presented in this paper obviously requires further testing on a larger number of samples. Nevertheless, it can be unequivocally stated that obtaining reliable, reference longitudinal wave speed, necessary to assess the scale of damage evolution in tomographic tests, must always be determined for concrete without damage of the same composition, and which is stored in the same conditions as the assessed concrete. In practice, this may be the maximal speed determined at the time of the test on the concrete structural member in a place where there are no defects, or on a sample without defects taken from the member. It should be emphasized in the light of outcomes of the tested beams for which only self-drying concrete and extending the period before the tomographic investigation by 7 days changed the reference speed by approximately 20%.

### 5.2. Prefabricated Beam

**Figure 23:**
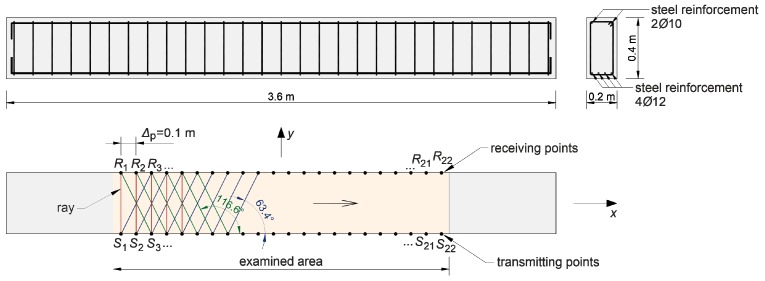
Scheme of the beam and the assumed system of transmitting/receiving points and rays.

**Figure 24:**
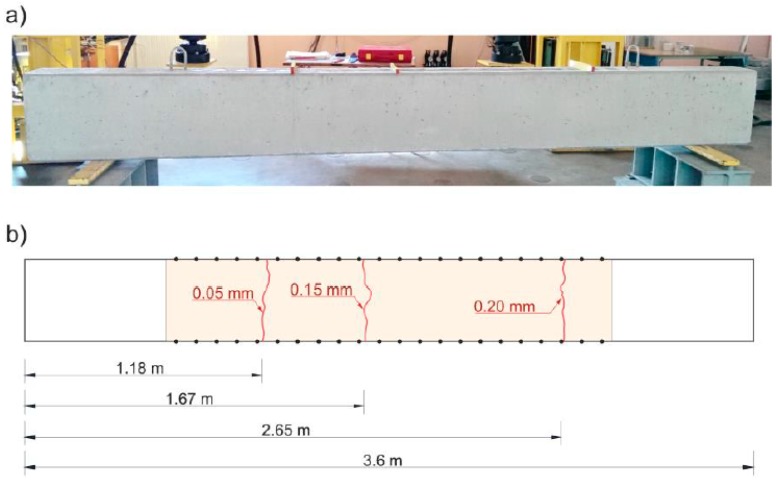
(**a**) Picture of the beam (the places of visible cracks are marked with the slats put on the upper surface of the beam). (**b**) Shape and width of the visible cracks.

**Figure 25:**
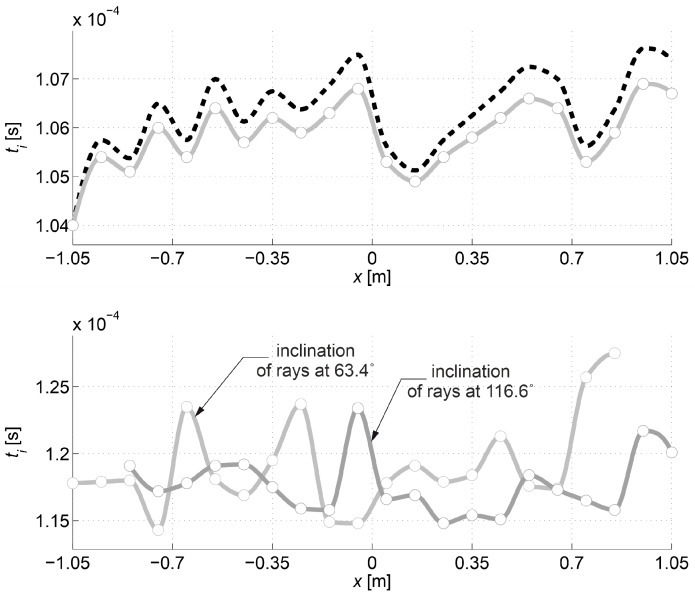
Propagation times $t_{\mathrm{path}\;\mathrm{int},i}$ and $t_{\mathrm{ray}\;\mathrm{approx},i}$ between the transmitting/receiving points in the beam. The results are presented as a function of the position of the transmitting points: $t_{\mathrm{path}\;\mathrm{int},i}$ grey lines with nodes marked with circles, and $t_{\mathrm{ray}\;\mathrm{approx},i}$ a black dashed line. The top diagram refer to the paths connecting the opposite points and the bottom one to the points lying diagonally at an angle of 63.4° and 116.6° to each other in relation to the axis x.

**Figure 26:**
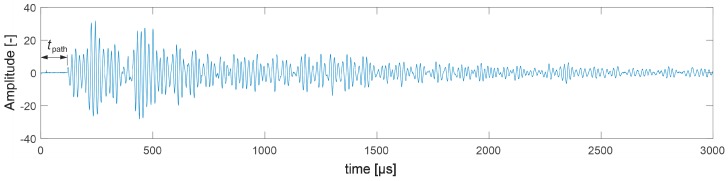
Example of a signal recorded by the receiving head (transmitting point No. 3 and receiving point No. 1).

A scheme of a beam is shown in Figure 23. Its dimensions were 20 cm ×40 cm × 360 cm and it was made of concrete with a mean compression strength after 28 days of $f_{\mathrm{cm},\mathrm{cube}}=38$ MPa. The maximal aggregate diameter was $d_{a\;\max}=16$ mm. The reinforcement was made of steel with characteristic yield point declared by the manufacturer of $f_{\mathrm{yk}}=500$ MPa. The longitudinal lower reinforcement consisted of four bars with a diameter of 12 mm, top reinforcement of two bars with a diameter of 10 mm and the transverse reinforcement was made of bi-armed stirrups with a diameter of 8 mm with a spacing of 125 mm. As mentioned at the beginning of this point, the beam was damaged during transport and there were three cracks crosswise to its axis, two of which were in the central area of the beam selected for tomographic imaging. The shape and width of this defects are shown in Figure 24. The studies consisted in the measurement of the time of propagation of the longitudinal ultrasound wave using a Pundit-Lab tester and transreceiver heads with a frequency of 54 kHz. The coupling of the heads and beam was provided by special gel for ultrasonic testing. Taking into account the measured longitudinal wave propagation times and basic frequency of the ultrasonic pulses, the average wavelength was ~7 cm and it allowed satisfying the basic requirements described in ASTM D2845-08 regarding the selection of frequency from the point of view of average grain size (as in point 5.1). The adopted system of transmitting/receiving points distant from each other by $\Delta_{p}=10$ cm is shown in Figure 23. Rays were assumed between the opposite points and those lying diagonally at an angle of ~26.6° and ~116.6° in relation to the beam axis (Figure 23). These angles have been changed from those used in computational examples and experiments on the laboratory beams to shorten to a reasonable minimum the length of diagonal paths taking account of the attenuation of ultrasound signals and to ensure the most correct reading of the longitudinal wave propagation times. In turn, the section for tomographic examinations was the vertical longitudinal plane of symmetry of the system running simultaneously between the longitudinal reinforcement bars. Measured and interpolated longitudinal wave propagation times $t_{\mathrm{path}\;\mathrm{int},i}$ are shown in Figure 25, where interpolated times are determined using a cubic Hermite spline. The diagrams also show $t_{\mathrm{ray}\;\mathrm{approx},i}$ determined according to relation (28) with $\beta =\beta_{\mathrm{opt}}$ according to formulas (24) and (25). On the other hand, Figure 26 shows an example of a recorded signal by the receiving head together with a reading of the time of longitudinal wave propagation. As in the case of the tests presented in point 5.1, Figure 26 shows first the signal caused by the propagation of the longitudinal wave and then the signal induced by the propagation of transverse and Rayleigh waves of much higher amplitude.

**Figure 27:**
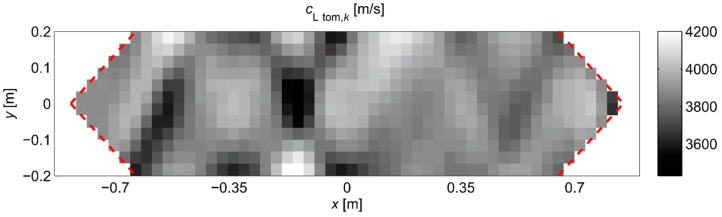
Tomographic reconstructions of the distribution of wave velocity according to Equation (3) in the longitudinal section of the beam (red dotted line—explanation in the text).

Figure 27 shows a tomographic reconstruction of the longitudinal wave velocity map in the longitudinal section of the beam which was determined by a randomized Kaczmarz method according to the information presented in point 2. The maximum measured average velocity of a longitudinal wave along all rays, i.e., 3913 m/s, was assumed to be $c_{L\;\mathrm{ref}}$. The resolution of $\delta_{1}\times \delta_{2}=3.33$ cm × 3.33 cm was applied and the arrangement of rays as in Figure 23 with addition of rays between real ones connecting fictitious transmitting/receiving points at a distance of every 6.25 mm. The results are shown only for the central section of the beam separated by a red dashed line through which all types of rays passed due to their inclination. The maps presented here are calculated on the basis of Equation (3) with propagation times $t_{\mathrm{ray},i}=t_{\mathrm{ray}\;\mathrm{approx},i}$ in accordance with (28) for the paths connecting opposite points and $t_{\mathrm{ray},i}=t_{\mathrm{path}\;\mathrm{int},i}$ for the diagonal paths. For this purpose, the values of $t_{\mathrm{path}\;\mathrm{int},i}$ are taken, as shown in Figure 25. The optimal coefficient β necessary for the determination of $t_{\mathrm{ray}\;\mathrm{approx},i}$ was calculated in accordance with formulas (24)–(25) and amounted to 0.25. At the same time $d_{\mathrm{cL}\;\mathrm{ray}\;\max}$ according to Equation (29) amounted to 0.007, which, in the considered case, proves the lack of significant influence of the generated cracks on the deflecting the fastest ultrasound wave propagation paths. This may also demonstrate the high degree of homogeneity of the concrete in the prefabrication plant.

In Figure 27, on the left side, clearly formed 2 elastically degraded zones can be seen which were created around the visible cracks (at x≈−0.6 m and x≈−0.15 m). In addition, there are also two other zones of this type which can be tomographically observed and were not signaled by visible defects (at x≈0.2 m and x≈0.5 m) and a few smaller ones, reaching up to about 6 ÷ 9 cm deep into the beam from its lower and upper surfaces. The latter may have been created before the beam was damaged as a result of shrinkage stresses occurring while the element was drying out after dismantling the beam formwork. Based on Equation (20), the maximal change of Young’s tangent modulus defined by the $\min\left(E_{D}/E_{0}\right)$ ratio at the 0.77 level can be estimated, if one assumes in this case that $c_{L0}=c_{L\;\mathrm{ref}}$ (in a zone that goes across the beam at x≈−0.15 m). The width of the defect at this point is approximately 17 cm. It is very close to the width of the elastically degraded zone, which was calculated theoretically in point 3. For the $\min\left(E_{D}/E_{0}\right)=0.77$ ratio, this corresponds to a damaged area width of approximately 18 cm, which can be read from Figure 5b or Figure 6b. This result also indirectly pre-confirms the validity of the identification method for the internal length $l_{c}$ of concrete proposed in point 3.

## 6. Conclusions

As a summary of this work, the following general conclusions should be highlighted:

(1) The accuracy of transmission ultrasonic tomography for the detection of brittle damage in concrete can be effectively supported by the graph theory and, in particular, by Dijkstra’s algorithm. What is important, it allows the determination of real paths of the fastest ultrasonic wave propagation in concrete containing localized elastically degraded zones at any stage of their evolution. Thanks to the analyses conducted on this basis, the authors developed the method of reducing errors in reconstructions of longitudinal wave speed maps. In this approach, the errors are decreased which are caused by using a simplification of straightness of the fastest wave propagation paths assumed in the typical mathematical apparatus for ultrasonic tomography. The method is based on the appropriate elongation of the measured propagation times of the wave travelling between opposite transmitting-receiving transducers if the actual propagation paths deviate from straight lines.

(2) Transmission ultrasonic tomography allows the estimation of the internal length of concrete defined in accordance with the methodology of damage mechanics. This problem is very important in the case of studies carried within this field of mechanics (e.g., Reference [29,30,31,49,50]) when predicting the extent and degree of brittle damage evolution in concrete structures. However, this conclusion may be addressed to testing RC beams with certain restrictions regarding issues of bonding between concrete and reinforcing bars and is appropriate for beams of lower reinforcement ratios with arrangement of tomographic rays not along reinforcing bars.

(3) Knowledge of the internal length of concrete allows rational determination of the appropriate resolution in ultrasonic tomography imaging and assessment of evolution of localized elastically degraded zones.

(4) The use of fictitious transmitting-receiving points in ultrasonic tomography, for which wave propagation times are calculated by interpolation of measured times, can contribute to the reduction of the required number of transducers and possible costs in the considered approach to concrete beams assessment while maintaining proper resolution of tomographic images. This outcome was well-grounded in the case of numerical analysis conducted in the work and usefulness of this approach was pre-confirmed in the own experiments. However, due to the limited number of these tests, it needs further justification and experimental studies using different arrangements of distances between sending-receiving points for ultrasonic pulses on the same RC members and confrontation with tests based on, e.g., acoustic emission or X-ray tomography.

## Figures and Tables

**Table 1:** Maximum relative changes in path length and wave propagation times when changing $\Delta_{n}$ in different defect evolution phases.

$\max{\left|\frac{t_{\mathrm{path},i}\left(\Delta_{n2}\right)-t_{\mathrm{path},i}\left(\Delta_{n1}\right)}{t_{\mathrm{path},i}\left(\Delta_{n1}\right)}\right|}_{i=1,2,\ldots I}$ [-]				
$\Delta_{n1}$, $\Delta_{n2}$ [mm]	$\min\;\left(E_{D}/E_{0}\right)$ [-]			
	0.9	0.8	0.6	0.2
50, 100	0.0035	0.0085	0.0154	0.0150
25, 50	0.0030	0.0054	0.0104	0.0230
12.5, 25	0.0024	0.0020	0.0030	0.0057
6.25, 12.5	0.0011	0.0012	0.0021	0.0040
3.125, 6.25	0.0003	0.0006	0.0004	0.0011
$\max{\left|\frac{L_{\mathrm{path},i}\left(\Delta_{n2}\right)-L_{\mathrm{path},i}\left(\Delta_{n1}\right)}{L_{\mathrm{path},i}\left(\Delta_{n1}\right)}\right|}_{i=1,2,\ldots ,I}$ [-]				
$\Delta_{n1}$, $\Delta_{n2}$ [mm]	$\min\;\left(E_{D}/E_{0}\right)$ [-]			
	0.9	0.8	0.6	0.2
50, 100	0.0199	0.0386	0.0794	0.0430
25, 50	0.0119	0.0245	0.0543	0.0281
12.5, 25	0.0071	0.0100	0.0262	0.0324
6.25, 12.5	0.0043	0.0072	0.0109	0.0180
3.125, 6.25	0.0016	0.0032	0.0067	0.0094

**Table 2:** $\beta_{\mathrm{opt}}$ calculated on the basis of data from Figure 11 in case of the opposite transmitting/receiving points in different phases of defect growth.

$\min\;(E_{D}/E_{0})$ [-]	0.9	0.8	0.6	0.2
$\beta_{\mathrm{opt}}$ [-]	0.09	0.15	0.33	1.24

**Table 3:** Global—mean square ($e_{g}$) and local—maximal ($e_{l\;\max}$) relative tomographic reconstruction error using the propagation times for the opposite points in Equation (3) $t_{\mathrm{ray},i}=t_{\mathrm{ray}\;\mathrm{approx},i}$ or $t_{\mathrm{ray},i}=t_{\mathrm{path},i}$. Maximal relative difference in average wave velocities over the rays $d_{\mathrm{cL}\;\mathrm{ray}\;\max\;}$ due to the use of $t_{\mathrm{ray},i}=t_{\mathrm{ray}\;\mathrm{approx},i}$ or $t_{\mathrm{ray},i}=t_{\mathrm{path},i}$.

$e_{g}$ [-]				
$t_{\mathrm{ray},i}$	$\min\;\left(E_{D}/E_{0}\right)$ [-]			
	0.9	0.8	0.6	0.2
$t_{\mathrm{ray}\;\mathrm{approx},i}$	0.001	0.004	0.011	0.027
$t_{\mathrm{path},i}$	0.002	0.005	0.018	0.087
$e_{l\;\max}$ [-]				
$t_{\mathrm{ray},i}$	$\min\;\left(E_{D}/E_{0}\right)$ [-]			
	0.9	0.8	0.6	0.2
$t_{\mathrm{ray}\;\mathrm{approx},i}$	0.007	0.022	0.058	0.186
$t_{\mathrm{path},i}$	0.010	0.025	0.099	0.700
$d_{\mathrm{cL}\;\mathrm{ray}\;\max}$ [-]				
$\min\;\left(E_{D}/E_{0}\right)$ [-]	0.9	0.8	0.6	0.2
Equation (27), Exact value	0.005, 0.009	0.016, 0.023	0.067, 0.068	0.325, 0.323

**Table 4:** Relative global interpolation errors of propagation times from the first example using a cubic Hermite spline in different phases of defect growth.

$\min\left(E_{D}/E_{0}\right)$ [-]	0.9	0.8	0.6	0.2
$e_{g}$ [-]	0.002	0.003	0.007	0.020

**Table 5:** $\beta_{\mathrm{opt}}$ calculated on the basis of data from Figure 14 in case of the opposite transmitting/receiving points in different phases of defect growth.

$\min\left(E_{D}/E_{0}\right)$ [-]	0.9	0.8	0.6	0.2
$\beta_{\mathrm{opt}}$ [-]	0	0	0.10	0.68

**Table 6:** Global—mean square ($e_{g}$) and local—maximal ($e_{l\;\max}$) relative tomographic reconstruction error using the propagation times for the opposite points in Equation (3) $t_{\mathrm{ray},i}=t_{\mathrm{ray}\;\mathrm{approx},i}$ or $t_{\mathrm{ray},i}=t_{\mathrm{path}\;\mathrm{int},i}$. Maximal relative difference in average wave velocities over the rays $d_{\mathrm{cL}\;\mathrm{ray}\;\max\;}$ due to the use of $t_{\mathrm{ray},i}=t_{\mathrm{ray}\;\mathrm{approx},i}$ or $t_{\mathrm{ray},i}=t_{\mathrm{path}\;\mathrm{int},i}$.

$e_{g}$ [-]				
$t_{\mathrm{ray},i}$	$\min\;\left(E_{D}/E_{0}\right)$ [-]			
	0.9	0.8	0.6	0.2
$t_{\mathrm{ray}\;\mathrm{approx},i}$	0.0018	0.0024	0.0083	0.0191
$t_{\mathrm{path}\;\mathrm{int},i}$	0.0018	0.0024	0.0100	0.0632
$e_{l\;\max}$ [-]				
$t_{\mathrm{ray},i}$	$\min\;\left(E_{D}/E_{0}\right)$ [-]			
	0.9	0.8	0.6	0.2
$t_{\mathrm{ray}\;\mathrm{approx},i}$	0.0108	0.0156	0.0467	0.1326
$t_{\mathrm{path}\;\mathrm{int},i}$	0.0108	0.0156	0.0598	0.5053
$d_{\mathrm{cL}\;\mathrm{ray}\;\max}$ [-]				
$\min\;\left(E_{D}/E_{0}\right)$ [-]	0.9	0.8	0.6	0.2
Equation (29) Exact value	0, 0.009	0, 0.023	0.021, 0.068	0.208, 0.323

**Table 7:** Cracking and maximal loads of the lab-beams.

Load	No. of the Beam			Mean
	1	2	3	
$P_{\mathrm{cr}}$ [kN]	27	30	28	28.3
$P_{\max}$ [kN]	43	45	46	44.7

**Table 8:** $\beta_{\mathrm{opt}}$ calculated on the basis of data from Figure 20 in case of the opposite transmitting/receiving points and $d_{\mathrm{cL}\;\mathrm{ray}\;\max}$ according to Equation (29) before and after an action of cracking load.

Optimal Scaling Factor β $\mathrm{and}\;d_{\mathrm{cL}\;\mathrm{ray}\;\max}\;\mathrm{Index}$	No. of the Beam		
	1	2	3
$\beta_{\mathrm{opt}}$ [-] before loading	0.74	1.34	0.73
$\beta_{\mathrm{opt}}$ [-] after action of cracking load	6.02	1.29	1.24
$d_{\mathrm{cL}\;\mathrm{ray}\;\max}$ [-] before loading	0.025	0.039	0.041
$d_{\mathrm{cL}\;\mathrm{ray}\;\max}$ [-] after action of cracking load	0.050	0.053	0.086
